# Engineering cell-free systems by chemoproteomic-assisted phenotypic screening†

**DOI:** 10.1039/d4cb00004h

**Published:** 2024-03-06

**Authors:** Zarina Levitskaya, Zheng Ser, Hiromi Koh, Wang Shi Mei, Sharon Chee, Radoslaw Mikolaj Sobota, John F. Ghadessy

**Affiliations:** a Protein and Peptide Engineering and Research Laboratory, Institute of Molecular and Cell Biology, Agency for Science, Technology and Research (A*STAR) 8A Biomedical Grove Singapore 138648 fghadessy@imcb.a-star.edu.sg; b Function Proteomics Laboratory, Institute of Molecular and Cell Biology, Agency for Science, Technology and Research (A*STAR) 8A Biomedical Grove Singapore 138648 rmsobota@imcb.a-star.edu.sg

## Abstract

Phenotypic screening is a valuable tool to both understand and engineer complex biological systems. We demonstrate the functionality of this approach in the development of cell-free protein synthesis (CFPS) technology. Phenotypic screening identified numerous compounds that enhanced protein production in yeast lysate CFPS reactions. Notably, many of these were competitive ATP kinase inhibitors, with the exploitation of their inherent substrate promiscuity redirecting ATP flux towards heterologous protein expression. Chemoproteomic-guided strain engineering partially phenocopied drug effects, with a 30% increase in protein yield observed upon deletion of the ATP-consuming SSA1 component of the HSP70 chaperone. Moreover, drug-mediated metabolic rewiring coupled with template optimization generated the highest protein yields in yeast CFPS to date using a hitherto less efficient, but more cost-effective glucose energy regeneration system. Our approach highlights the utility of target-agnostic phenotypic screening and target identification to deconvolute cell-lysate complexity, adding to the expanding repertoire of strategies for improving CFPS.

## Abstract 2

Phenotypic screening identified drugs capable of improving the yeast lysate cell-free protein synthesis (CFPS) yield. A drug target gene identified using mass spectrometry proteomics was deleted to yield a yeast strain with higher productivity CFPS.

## Graphical abstract

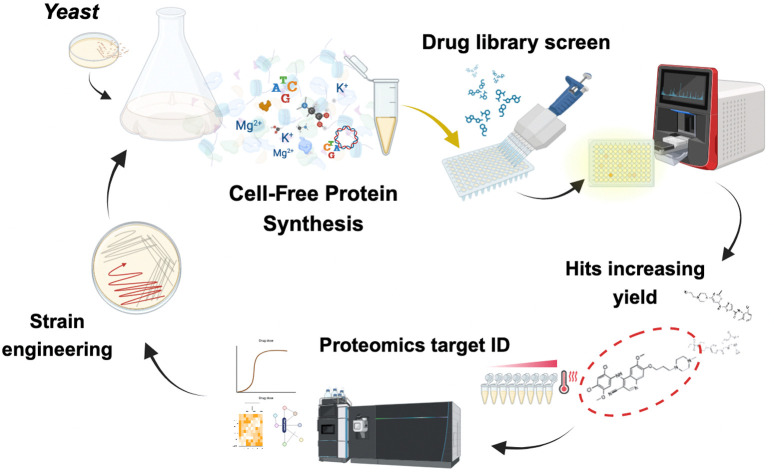

## Introduction

Phenotypic discovery approaches utilise compound or protein libraries to screen for desired phenotypes in living cells.^1–3^ As these are typically both mechanism and target agnostic, new biology can be discerned from hits illuminating the cellular pool of “dark biological matter”.^1,3^ Consequently, many hits from phenotypic screens would not have been readily discovered using target-based approaches.^1,4^ Here, we apply phenotypic screening towards engineering of poorly understood biologically complex cell-free protein synthesis (CFPS) systems. CFPS utilises cell-derived lysates supplemented with energy sources, amino acids, buffer components, nucleotides and nucleic acid template(s) encoding protein(s) of interest. Applications range from research-driven functional genomics, exploratory biomolecule and metabolic engineering, directed evolution, biosensing, diagnostics, and bio-circuit development, to large-scale industrial manufacturing of biopharmaceuticals and value-added compounds.^5–15^ Compared to cellular expression, the improved *in vitro* reaction tractability has driven rapid evolution of CFPS, increasing cost-efficiency and expanding its repertoire of products.^16^ These now include difficult-to-synthesize proteins such as antibodies, membrane proteins, proteins incorporating unnatural amino acids, and virus-like particles.^8,17–25^ Nevertheless, efficient complex protein production and high costs of exogenous reaction components such as energy factors, amino acids, tRNAs and nucleotides remain major challenges of CFPS development.^7,8,16,26^ These have been addressed using mainly rational approaches including template and strain engineering, growth condition modulation, development of alternate energy regeneration systems and systematic reaction component optimization.^27–38^ The CFPS system with the greatest productivity to date exploits *E. coli* lysates capable of synthesizing proteins at mg mL^−1^ scale.^39^ Prokaryotic lysates are not however inherently suitable for the production of post-translationally modified eukaryotic proteins, requiring the introduction of accessory enzymes and exogenous structures such as microsomes for complex protein synthesis.^6,35,40,41^ Whilst eukaryote-derived lysates can overcome these issues, they typically show significantly reduced overall productivity, in part associated with their complex regulatory networks and increased background ATP consumption.^42–45^ A case in point is lysates derived from *S. cerevisiae*, an organism historically known for its wide application in biocatalysis and biomanufacturing.^45,46^ Whilst yeast lysates can outperform other eukaryotic CFPS systems^47^ (particularly rabbit reticulocyte lysates) they still remain amongst the least productive, and there is a need for continuing optimisation.^8,48^

Proteins made by CFPS are readily quantifiable, rendering phenotypic screening an ideal tool to interrogate small molecule libraries for modulators of protein expression. Using this approach, we identified several compounds from a library of FDA-approved drugs that increased heterologous protein expression in *S. cerevisiae* cell lysates. Notably, many of these were ATP-mimetic kinase inhibitors, suggesting that inhibition of endogenous energy consumption pathways channeled more ATP into protein synthesis. To further validate this hypothesis, we used thermal stability proteomics analysis^49^ for the first time in CFPS to identify novel protein targets that potentially compete for ATP. Lysates prepared from a yeast strain deleted for one of these targets, the ATP-consuming SSA1 component of the HSP70 chaperone, were more productive in CFPS.

## Results and discussion

### Screening of an FDA-approved small molecule library for CFPS agonists

**Fig. 1:**
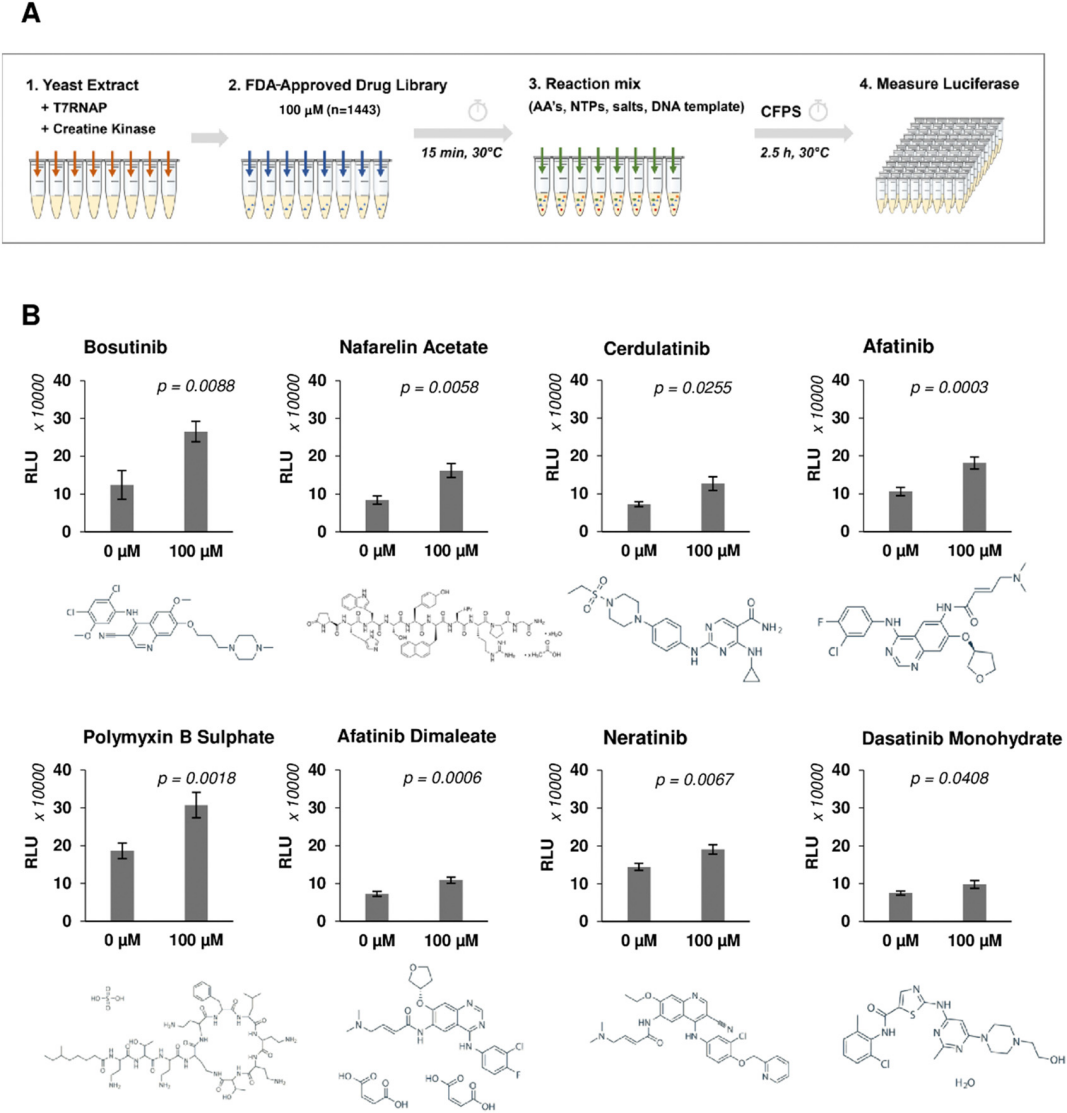
Yeast lysate CFPS phenotypic screening workflow to identify compounds that increase heterologous protein expression yield. (A) Schematic of the drug screening workflow. Drugs (100 μM, *n* = 1443) were incubated with yeast extract for 15 minutes prior to addition of nLuc DNA template and other essential components (amino acids, nucleotides, salts) to initiate expression. nLuc produced was measured after 2.5 h incubation. (B) Repeat experiment of selected drug candidates showing fold increase in nLuc production. *n* = 3 ± SD. Significance measured using two-tailed Student's *t*-test.

A high-throughput screen was performed using a library of 1443 FDA-approved drugs on yeast CFPS to identify enhancers of heterologous protein production (Fig. 1(A)). Coupled transcription and translation reactions were set up according to optimised protocols.^48,50^ Translation was initiated *via* a cap-independent mechanism by the inclusion of the TMV virus 5′UTR Ω sequence upstream of the nano-luciferase (nLuc) reporter gene, and transcripts were stabilised by the addition of a 90-nt poly-A tail at the 3′ end.^50^ Yeast extract fractions were pre-incubated with drugs for 15 min, allowing sufficient time for binding and onset of drug-mediated responses. Reactions were initiated by the addition of DNA template, amino acids, nucleotides, and energy factors. Drug candidates were identified based on the fold increase of luminescence signal observed from drug-treated reactions against drug-free controls and a 50% cut-off was applied to select hits for secondary analysis (Fig. S1, ESI†). Repeat experiments confirmed 8 out of 110 hits, increasing nLuc yields by 1.30–2.13-fold (Table 1 and Fig. 1(B)). None of the positive hits were shown to directly enhance nLuc activity in a counter-screen to exclude false positives (Fig. S2A, ESI†). Interestingly, six out of eight hits (bosutinib, cerdulatinib, afatinib, afatinib dimaleate, neratinib and dasatinib) are tyrosine kinase inhibitors (TKIs) known to compete for ATP-binding sites of various targets including SRC, ABL, JAK, and EGFR.^51–55^ The remaining two are the gonadotropin-releasing hormone agonist nafarelin acetate and the antibiotic polymyxin B sulphate, which disrupts Gram-negative bacterial membranes.^56,57^

**Table 1:** List of drug hits increasing yeast extract productivity

Drug	Mode of action	Targets	
Bosutinib	ATP-competitive tyrosine kinase inhibitor (BCR-ABL/SRC)	Tyrosine kinases	BCR, ABL1, LYN, HCK, SRC
		Serine/threonine kinases	CDK2, MAP2K1, MAP2K2, MAP3K2, CAMK2G
		ATP-dependent efflux pump	ABCB1
Cerdulatinib	ATP-competitive tyrosine kinase inhibitor (SYK/JAK)	Tyrosine kinases	SYK, JAK1/2/3, TYK2
Dasatinib monohydrate	ATP-competitive tyrosine kinase inhibitor (SRC family)	Tyrosine kinases	ABL1/2, SRC, EPHA2/5, LCK, YES1, KIT, PDGFRB, FYN, BTK, BCR, CSK, EPHB4, FGR, FRK, LYN
		Serine/threonine kinases	ZAK, MAPK14,
		Transcription factors	STAT5B, NR4A3,
		Heat shock protein	HSPA8
		Phosphoribosyltransferase	PPAT
		ATP-dependent efflux pump	ABCB1
		ATP-binding cassette	ABCG2
Nafarelin acetate	Gonadotropin-releasing hormone (GnRH) agonist	G-protein-coupled receptors	GNRHR
Afatinib	ATP-competitive tyrosine kinase inhibitor (ErbB family)	Tyrosine kinases	EGFR, ERBB2/4
		ATP-dependent efflux pump	ABCB1
		ATP-binding cassette	ABCG2
Neratinib	ATP-competitive tyrosine kinase inhibitor (HER2/EGFR)	Tyrosine kinases	EGFR, HER2
		Serum proteins	ALB, AAG
		ATP-dependent efflux pump	ABCB1
Polymyxin B sulphate	Antibiotic	LPS of Gram-negative bacteria outer membrane	Displaces Ca^2+^ and Mg^2+^ from LPS increasing membrane permeability

Numerous drugs which reduced the nLuc yields were also identified in the screen (Fig. S3 and Table S1, ESI†). None were direct inhibitors of nLuc (Fig. S2B, ESI†). These comprised known protein synthesis inhibitors (hygromycin B, puromycin, gentamicin, pentamidine and paramomycin), thus validating screen efficiency. Other inhibitors likely impede the transcriptional step (doxorubicin, proflavine hemisulfate), interfere with DNA synthesis, induce DNA damage (aprotonin, ethacridine lactate and mitoxantrone) or inhibit T7RNAP (peparin).^58,59^ The remaining two inhibitory compounds, calcium levofolinate (chemotherapy adjuvant) and carbenoxolone sodium (antiulcer agent), are of further potential interest.

### Additive effects of drugs enhancing CFPS

**Fig. 2:**
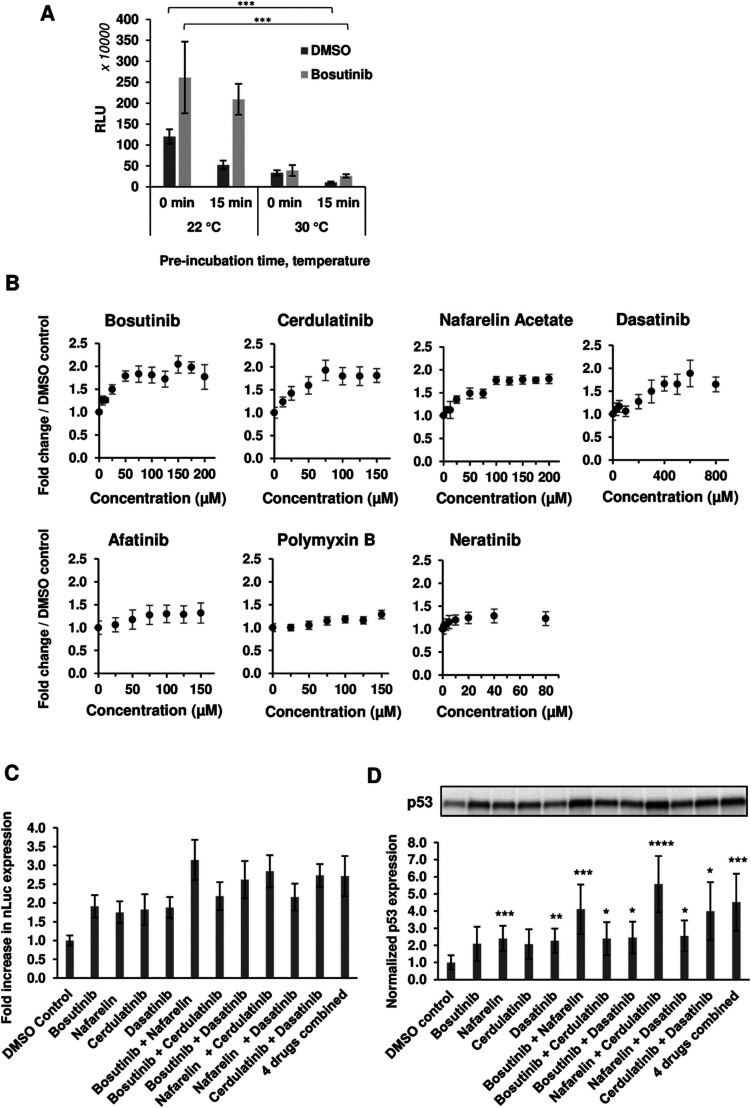
Optimization of the drug incubation conditions. (A) Effect of CFPS temperature and drug pre-incubation was assessed on the final nLuc yield. A reaction mix containing nLuc template was added to the yeast extract fraction either directly with 100 μM bosutinib/vehicle or after 15 min pre-incubation of the extract. Extract pre-incubation and subsequent CFPS were performed either at 22 °C or 30 °C. *n* = 3 ± SD. *p < 0.05; **p < 0.01; ***p < 0.005 (two-tailed Student's *t*-test). (B) nLuc expression in yeast lysate measured over the indicated drug concentrations. *n* = 3 ± SD. (C), (D) Bosutinib, cerdulatinib, nafarelin acetate and dasatinib were added to CFPS reactions at their optimum concentrations individually or at the indicated combinations. The drug effect was assessed on the synthesis of nLuc (C) and p53 (D) by means of luminescence and western blot densitometry analysis, respectively. All readings were normalised to DMSO-treated controls. *n* = 3 ± SD.

We next varied the reaction conditions to see if drug activity could be further potentiated. Significant improvements in total yields were observed with lower reaction temperature (22 °C *versus* 30 °C) and removal of the drug pre-incubation step prior to initiation of transcription/translation (Fig. 2(A)). The pre-incubation period (15 min) reduced the extract activity at both temperatures, likely due to competing background reactions and reduced viability (particularly at the higher incubation temperature) prior to the initiation of luciferase expression by the addition of template and energy factors. The observed inhibition could be rescued with drug treatment (Fig. 2(A)). Following this modified protocol, dose responsive increases (1.28–1.95-fold) in nLuc yield were observed, further validating hits (Fig. 2(B)). We next tested drug combinations for possible additive/synergistic effects. Four of the most potent drugs, bosutinib, cerdulatinib, nafarelin acetate and dasatinib, were added to CFPS reactions alone or in combination. Combining nafarelin with either cerdulatinib or bosutinib exhibited a partial additive effect, further enhancing nLuc production from the original 1.75–1.91-fold to 2.85–3.15-fold (Fig. 2(C)).

Interestingly, the minimum additive effect was observed when combining bosutinib with cerdulatinib and nafarelin acetate with dasatinib, suggesting possible overlapping functions of the drugs within pairs. A similar pattern was observed with *in vitro* expression of the p53 test protein, whereby the drugs resulted in a 1.98–2.40-fold increase in yield when added alone and as high as 3.88 and 5.47-fold when nafarelin acetate was respectively combined with either bosutinib or cerdulatinib (Fig. 2(D)).

### Exploring the functional role of bosutinib in improving yeast CFPS

**Fig. 3:**
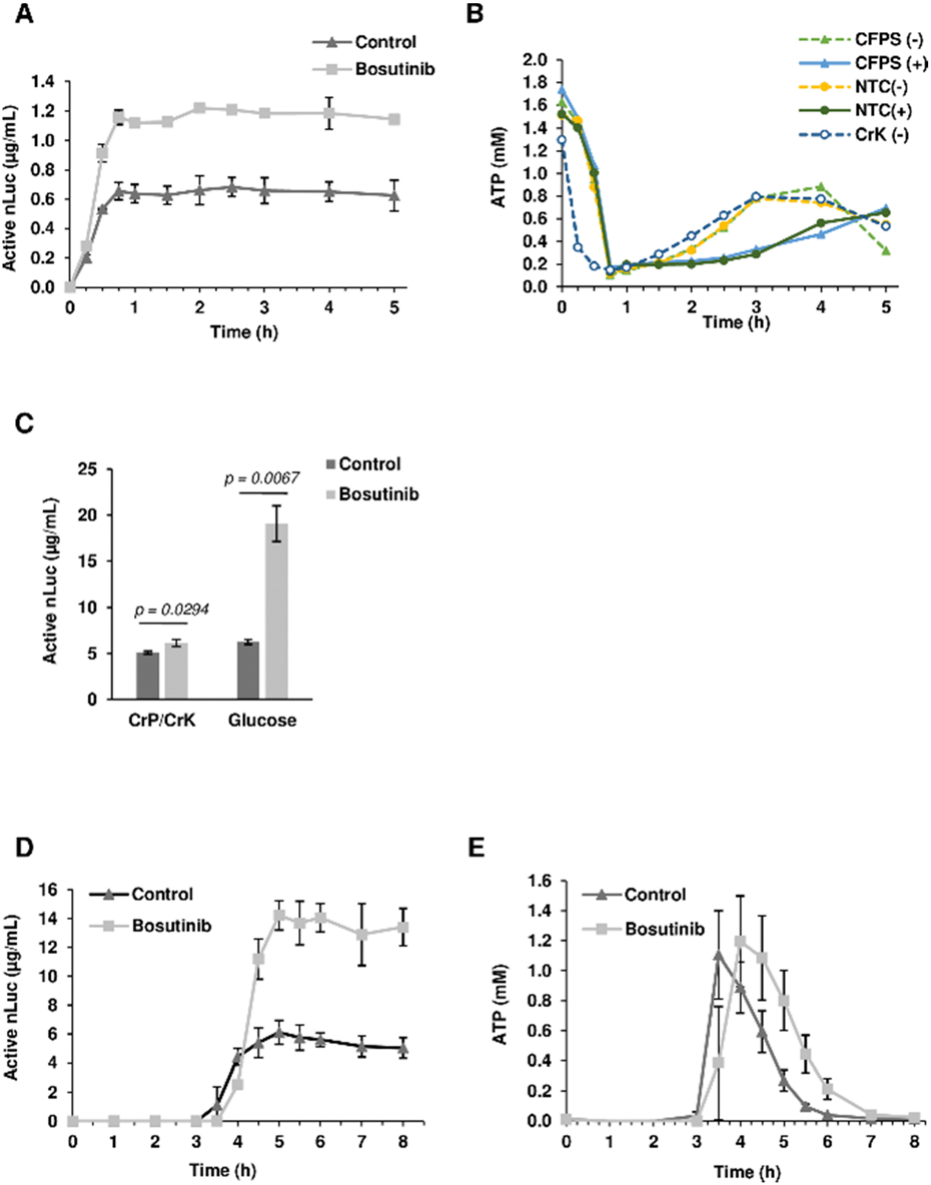
Characterization of the bosutinib effect on yeast CFPS. (A) nLuc synthesis in CFPS reactions treated with 0 μM and 100 μM bosutinib was monitored at varying time intervals from 0–5 h. *n* = 2 ± SD. (B) Corresponding ATP levels were measured from nLuc-synthesizing CFPS reactions, NTC controls (−/+ indicating addition of bosutinib) and CFPS reactions without the creatine phosphate secondary energy regeneration system. (C) Productivity of the yeast extract from cells harvested at OD_600_ = 1.2 was assessed. CFPS reactions were treated with 100 μM bosutinib and set with either creatine phosphate or glucose as a secondary energy source. *n* = 3 ± SD. (D,E) nLuc and ATP levels in glucose-driven reactions were monitored from 0–8 h. *n* = 2 ± SD.

Given its role as an ATP-competitive kinase inhibitor, we questioned whether bosutinib (one of the best hits) increases the CFPS yield by reducing background ATP consumption. More ATP could then be funneled into heterologous protein synthesis, particularly for tRNA aminoacylation. Time-course experiments revealed that the majority of protein synthesis (>90%) occurred within the first 30 min, reaching completion by 45 min (Fig. 3(A)). Bosutinib did not prolong the reaction time, but almost doubled the rate of synthesis in the period between 15–30 min. Completion of the reaction can be explained by rapid depletion of ATP to almost 0.2 mM after 45 min (Fig. 3(B)). No evident differences in ATP consumption were observed between bosutinib treated and untreated reactions. Moreover, the rates of ATP consumption in reactions actively synthesizing nLuc were comparable to controls (no DNA template encoding nLuc added), indicating a minimal effect of nLuc synthesis on overall ATP consumption (Fig. 3(B)). Nevertheless, the addition of bosutinib did result in altered ATP levels both in CFPS and NTC control reactions, but at a much later time. Bosutinib either suppressed or delayed regeneration of ATP that occurred after 2 hours (Fig. 3(B)). Moreover, the observed regeneration of ATP must have been driven by endogenous processes, as it also occurred in CFPS lacking exogenous creatine phosphate and creatine kinase added to generate ATP (Fig. 3(B)). Yeast extracts have the capacity to generate ATP *via* the glycolytic pathway, with the addition of glucose, cAMP and inorganic phosphate facilitating the synthesis of 3.64 μg mL^−1^ of active luciferase.^60^ ATP regeneration *via* the glycolytic pathway occurred with a delay after 1.5 hours, with subsequent protein synthesis initiated at around 2 h.^60^ We thus tested if bosutinib's effect on ATP levels observed in the later time period would also impact protein synthesis in glucose-driven CFPS. Interestingly, bosutinib exhibited a 3.5-fold increase in nLuc yield, attaining a similar productivity level to creatine phosphate/kinase-driven CFPS (Fig. S4A, ESI†).

The absolute yield of active nLuc produced using the creatine phosphate/kinase energy system was 0.77 μg mL^−1^ and increased to 1.65 μg mL^−1^ upon treatment with bosutinib. Despite using a near-identical preparation protocol, the efficiency of our base yeast cell extract was far less than the reported 7 μg mL^−1^.^48^ Drastic drops in efficiency have been reported when extracts are prepared from cells harvested at the stationary phase due to metabolic shifts in response to stress and nutrient depletion.^30^ Our use of standard Erlenmeyer flasks as opposed to Tunair flasks could have resulted in accumulated stress due to prolonged culture time to reach the desired OD600 of 12.^30^ For subsequent experiments, extracts were therefore prepared from yeast harvested much earlier at OD600 of 1.2. These extracts were more efficient, and alongside optimization of template concentration, yielded 5.09 and 6.27 μg mL^−1^ of active nLuc with the creatine phosphate/kinase and glucose energy regeneration systems, respectively (Fig. 3(C) and Fig. S4B, ESI†). Notably, bosutinib lead to a greater increase in yield when the glucose energy regeneration system was used (Fig. 3(C)). The 3-fold increased nLuc yield to 19 μg mL^−1^ is the highest thus far reported using the cost-effective glucose energy alternative.^60^ Interestingly, a time-course experiment with glucose-driven reactions supported the hypothesis of bosutinib's effect on ATP consumption. As before, bosutinib increased the rate of nLuc production (Fig. 3(D)), and reactions containing bosutinib were able to sustain higher levels of ATP in the 4–6 hour time period post initiation (Fig. 3(E)). Moreover, the significantly later onset of nLuc production in glucose-driven reactions (3 hours compared to immediate initiation for creatine phosphate driven reactions) (Fig. 3(A) and (D)) could explain differences observed in yield between the two energy systems when the DNA template concentration was increased. Similar to previous studies, saturation in yield was observed with 10 nM of template using creatine phosphate as an energy source.^48^ However, with glucose-based energy regeneration, an optimal template concentration of 40 nM increased the nLuc yield by 7-fold (Fig. S4B, ESI†). Here, increased template degradation by endogenous nucleases during the considerable 3 hour lag phase is likely mitigated by increasing input DNA levels.

### Exploring drug effects on HeLa cell-based CFPS

**Fig. 4:**
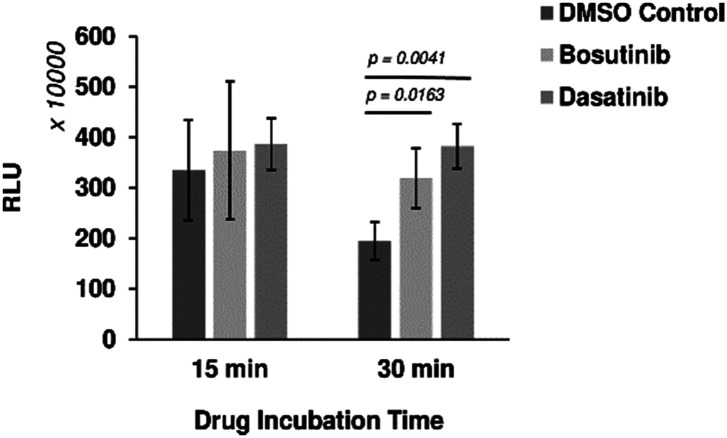
Drug effects on mammalian cell-based CFPS. Bosutinib and dasatinib monohydrate were pre-incubated with the HeLa cell lysate fraction for 15 or 30 min. Subsequent nLuc production was assessed with respect to DMSO-treated controls. *n* ≥ 3 ± SD.

Six out of the seven positive hits identified in the screen were drugs designed to target human cells. We therefore investigated if the observed outcomes could be translated to the improvement of more complex eukaryotic CFPS systems. HeLa cell-based CFPS reactions were tested with the compounds for improved nLuc production. No significant increase in yield was observed using the original drug screening protocol (15 min incubation with core extract prior to addition of the template and energy components), but following prolonged pre-incubation of the extract fraction with the drug (30 min), the yields of reactions containing bosutinib and dasatinib were 1.64 and 1.97-fold higher than the corresponding controls with DMSO (Fig. 4). However, the overall yields did not improve with the 30 minute pre-incubation time compared to non-treated lysates with 15 minute pre-incubation time. During the prolonged incubation, increased background depletion of endogenous ATP (up to 5 mM) will occur. This reduction in total ATP levels (endogenous + regenerated) is likely responsible for the observed drug-effects, as inhibition of competing ATP consumers will not be as beneficial when ATP is not limiting. A similar drug effect was observed for yeast lysates with and without pre-incubation (Fig. 2(A)), highlighting bosutinib and dasatinib as useful tools to suppress background ATP metabolism during the preparation of eukaryotic CFPS systems and improve efficiency.

### Thermal proteomics approach for target identification

**Fig. 5:**
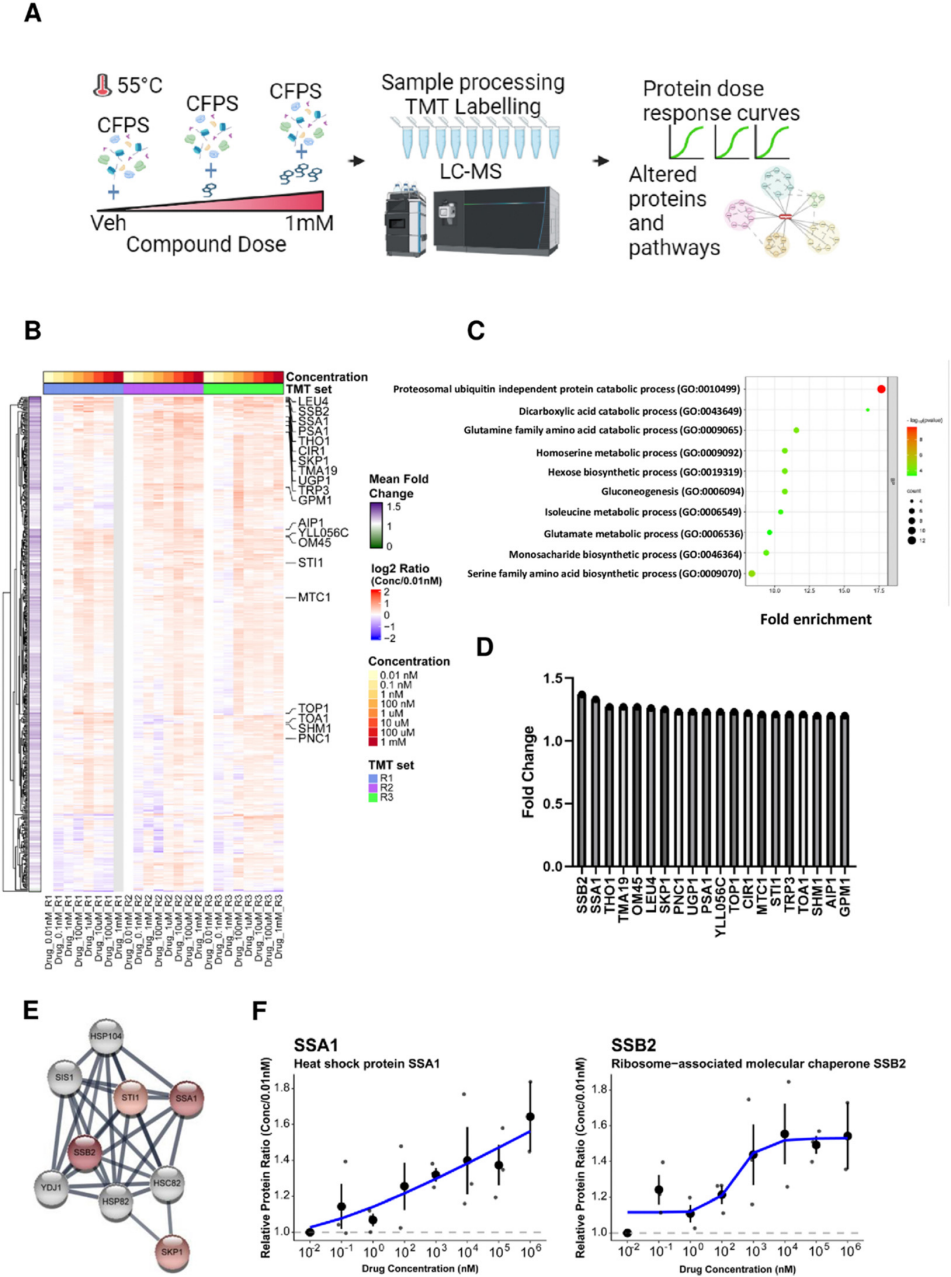
Effect of bosutinib on proteins and pathways in CFPS identified by thermal proteomics. (A) Workflow for thermal proteomics on the cell free protein system. Yeast extracts were treated with different doses of bosutinib (vehicle, 0.01 nM, 0.1 nM, 1 nM, 100 nM, 1 μM, 10 μM, 100 μM and 1 mM) and subjected to heat stress at 55 °C. Proteins from the different conditions were processed and peptides were labelled using tandem mass tags (TMT). Liquid chromatography coupled with tandem mass-spectrometry (LC–MS/MS) acquisition was performed to profile proteins based on the dose response relationship. Data analysis was carried out to identify proteins and pathways affected by bosutinib treatment. (B) Heat map showing the relative protein changes normalized to the lowest drug concentration (0.01 nM) across 457 proteins identified from three TMT replicate experiments. Twenty proteins with greater than 1.2-fold increase in mean fold change are annotated in the heatmap. (C) Top 10 biological processes based on gene ontology (GO) enrichment analysis using 231 proteins with mean increase ≥10%. (D) Bar plot of the twenty proteins with greater than 1.2-fold increase in decreasing order of magnitude. SSB2 and SSA1 were identified as the top 2 proteins with mean fold change of 1.37 and 1.33, respectively. (E) Protein interaction network for the top 20 hits highlighted four chaperone proteins (SSB2, SSA1, SKP1, STI1) using the STRING database (proteins are coloured in red based on mean fold change with the color intensity reflecting a higher mean fold-change). (F) Individual loess curves fitted for drug dose against protein quantity measured by mass spectrometry for SSA1 and SSB2, respectively.

Thermal stability-based proteomics methods such as the cellular thermal shift assay (CETSA)^49,61^ or thermal proteome profiling (TPP)^62–64^ coupled to mass spectrometry (MS) can identify proteins whose thermal stability is affected by small molecule action. In particular, isothermal dose response (ITDR) analysis allows the identification of dose-dependent effects on protein thermal stability at elevated temperatures,^65^ enabling CETSA application to a variety of cellular systems for identification of possible targets of small molecules.^66–68^ Yeast extracts were treated with bosutinib at 9 different concentrations (0.01 nM to 1 mM) along with a vehicle control prior to heat treatment at 55 °C. Proteins stabilized by bosutinib binding at this elevated temperature were then quantified by tandem mass tag (TMT) labelling, and dose response curves and pathway analysis were performed (Fig. 5(A)). A total of 545 proteins were fully quantified from the treated yeast lysates across the three replicates. Proteins with high coefficient-of-variation (%CV) greater than 20% at the lowest and highest three drug concentrations were filtered away, leaving 457 proteins which were quantified. Relative protein ratios were computed by normalizing against the lowest concentration (0.01 nM), and mean fold-changes were obtained by dividing the mean protein abundance in the highest three concentrations against the mean of the lowest three concentrations across the three replicates (Supplementary File 1, ESI†).

Two hundred and fifty one proteins were identified with at least 10% increase in protein abundance in the presence of bosutinib (Fig. 5(B)). Of these, the cytosolic HSP70 chaperone proteins SSA1 and SSB2 were the top 2 proteins stabilised, with up to ∼1.6-fold dose-responsive increases in abundancy measured (Fig. 5(D) and (F)). Furthermore, known interactors of SSA1 and SSB2 (SKP1 and STI1) are present within the top 20 drug-stabilised proteins, strongly implicating the ATP-consuming HSP70 chaperones as bosutinib targets (Fig. 5(E)). Gene set enrichment analysis of stabilised proteins identified 77 significantly enriched biological pathways. The most enriched pathways included proteasomal ubiquitin-independent proteins (PRE1, PRE3-9, PUP1-3) and other metabolic pathways, such as dicarboxylic acid, glutamine, homoserine, hexose and gluconeogenesis metabolism (Fig. 5(C)). Several components of these pathways utilize ATP (*e.g.* proteasome complex, PCS60, THR1, PCK1 and PYC2), and non-specific inhibition by bosutinib may contribute to reducing background lysate ATP metabolism. We additionally performed a proteomics time-course experiment at 37 °C to monitor protein stability during a CFPS reaction (Fig. S5 and Supplementary File 1, ESI†). The SSA1 and SSB2 protein levels remained consistent, indicating that the dose-dependent stabilising effects of bosutinib were not confounded by intrinsic thermal instability.

### Chemoproteomic-guided strain engineering to improve CFPS

**Fig. 6:**
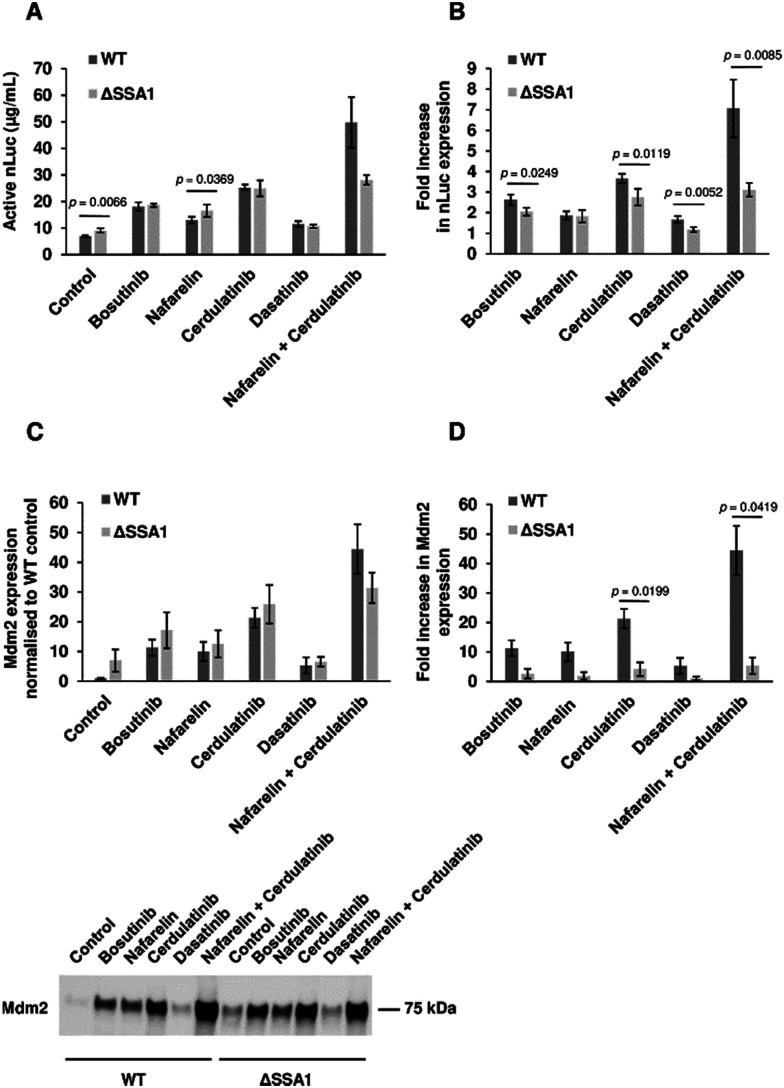
Chemoproteomic-guided strain engineering. (A) Activity of extracts from BY4743-WT and BY4743-Δ*SSA1* strains (without drug treatment and with bosutinib, nafarelin acetate, cerdulatinib and dasatinib) were compared in nLuc expressing CFPS reactions set using glucose as the secondary energy source. *n* = 4 ± SD. *p* values determined by two-tailed Student's *t*-test. (B) Drug effects on nLuc synthesis in CFPS reactions using WT and Δ*SSA1* extracts were compared by determining the fold increase in yield with respect to corresponding drug-free controls. *n* = 4 ± SD. *p* values determined by two-tailed Student's *t*-test. (C), (D) CFPS reactions with WT and Δ*SSA1* extracts were set for Mdm2 protein synthesis. Protein yields were assessed using densitometry analysis of the western blot image. The Mdm2 yield in the Δ*SSA1* extract is normalised to that of the WT extract (C top). Representative blot of Mdm2 synthesized by drug treated extracts is presented (C bottom). Fold increase in Mdm2 levels is calculated with respect to the corresponding WT and Δ*SSA1* controls (D). *n* = 2 ± SD. *p* values determined by two-tailed Student's *t*-test.

The BY4743-*SSA1*Δ strain from the homozygous knockout collection was selected to investigate if single gene knockout could reproduce drug-mediated enhancement of yeast CFPS. Extracts from BY4743 WT and *SSA1*Δ strains were prepared as previously described. As reported earlier, deletion of the *SSA1* gene displayed no effect on cell growth^69^ (Fig. S6, ESI†). Glucose-driven CFPS reactions produced 30% more nLuc (9.1 μg mL^−1^) when *SSA1*Δ extract was used (Fig. 6(A)). Moreover, the addition of bosutinib resulted in significantly greater improvement of the WT extract (2.6-fold) than the *SSA1*Δ extract (2.0-fold). A similar pattern was observed with two other TKI drug hits (cerdulatinib and dasatinib), but not with nafarelin acetate GnRH agonist (Fig. 6(B)). We further treated lysates with drug combinations previously shown to improve yield (Fig. 2(C)). Up to 49.7 and 28.1 μg mL^−1^ of nLuc was made using combined cerdulatinib and nafarelin treatment in parental and *SSA1*Δ strains, respectively. A very similar pattern was observed when comparing the expression yields of another test protein, the E3 ligase Mdm2, in parental and *SSA1*Δ extracts. In the absence of drugs, the yields were higher in the *SSA1*Δ extract (Fig. 6(C)). Drug addition also resulted in less improvement of the yield when added to the *SSA1*Δ extract (Fig. 6(D)). Notably, the expression yield of Mdm2 could be improved up to 44 times using the nafarelin and cerdulatinib combination (Fig. 6(C)).

Our study demonstrates the utility of phenotypic screening to increase the productivity of CFPS. High-throughput screens have been coupled with CFPS technology for protein interaction studies, prototyping, directed evolution and identification of novel therapeutics.^27,70–72^ To date, none have been designed to improve the translation efficiency of the CFPS system itself. The majority of hit compounds identified in the screen were tyrosine kinase inhibitors, suggesting redirection of ATP flux towards the protein translation machinery, particularly ATP-dependent tRNA synthetases. This assumption was verified for the ATP-competitive kinase inhibitor bosutinib, where heterologous protein expression increased with no overall change in extract ATP consumption kinetics. Thermal stability-based proteomic analysis of yeast extracts treated with bosutinib identified proteins involved in metabolic activity, some of which are ATP-dependent enzymes as possible targets. Whilst proteomics approaches have been used to characterize protein composition and quantities in CFPS for evaluation purposes,^73–75^ they have not previously been employed to delineate competing endogenous pathways and guide strain engineering. The most likely drug target candidates identified are the HSP70 molecular chaperones SSB2 and SSA1. HSP70 chaperones bear an ATPase domain involved in the regulation of substrate binding and are highly expressed.^76–78^ Deletion of the yeast *SSA1* gene resulted in 30% improvement of active nLuc synthesis, partially phenocopying bosutinib treatment of parental lysate. Furthermore, bosutinib showed reduced potentiation of *SSA1*Δ extracts, further validating SSA1 as a target. Notably, two other TKI hits (cerdulatinib and dasatinib) also showed reduced efficacy in *SSA1*Δ extracts, suggesting SSA1 to be a promiscuous substrate for ATP analogues. Nevertheless, SSA1 is not the sole target of identified TKI's given the high homology among the 4 SSA isoforms (SSA1-4) and 2 SSB isoforms (SSB1-2) of HSP70 chaperones.^76^ SSA1 is orthologous to several human heat shock proteins (HSPA1L, HSPA1B, HSPA8), highlighting these as potential ‘off-targets’ mediating the increased expression yield in HeLa cell lysates treated with bosutinib. Other potential ATP-consuming off-targets of bosutinib include SRC, ERK1/2 and JNK.^79^ Mass-spectrometry-based phosphoproteomics is an ideal complementary tool to further explore both of these and delineate further competitors and associated pathways.^80^ Strain engineering to delete further candidate ATP-metabolising targets will likely pose host cell viability issues that can be circumvented by our drug supplementation approach and/or lysate depletion of genomically tagged target proteins.^81^

The most efficient cell extracts to date are derived from *E. coli*, typically yielding 0.1–3 mg mL^−1^ of heterologous proteins.^8^ However, these are largely incapable of complex protein synthesis and post-translational processing (*e.g.* glycosylation) required for functional eukaryotic proteins. Whilst the use of alternative cell extracts derived from higher eukaryotes can address this deficit, these typically generate lower protein yields. Furthermore, they are associated with increased cost of production due to additional processing steps and overall less robust cell growth conditions.^82^ Microbial eukaryotes such as *S. cerevisiae* and *P. pastoris* are potentially ideal organisms to derive cell-free extracts from. They convey advantages of easy genetic manipulation and propagation.^83^ However, CFPS systems derived from these organisms are much less productive than *E. coli*-based lysates. *P. pastoris* CFPS can yield 50–300 μg mL^−1^ of protein, with maximal yields in *S. cerevisiae* trailing at 20 μg mL^−1^.^84–86^ The latter has been obtained after significant efforts to boost yields and overall cost efficiency using numerous approaches.^30,50,60,87^ Key strategies involved fine-tuning of extract metabolic processes through optimization of growth and harvesting conditions and elimination of proteins with inhibitory effects on CFPS such as PEP4 protease, XRN1 nuclease and STM1 ribosome inactivation factor.^30,44,50,84^ Our approach further increased nLuc yield 7-fold to 50 μg mL^−1^, the highest reported so far in yeast, and adds to the growing compendium of CFPS components and parameters that can be tuned to improve performance.

Recent advances in sugar-based energy regeneration systems hold great promise for widespread adoption of CFPS in academia and industry.^26,33,60,88^ These have driven down costs of yeast CFPS, despite the reduced productivity (3.6 μg mL^−1^ nLuc) compared to the widely-adopted and more costly creatine phosphate/creatine kinase system for ATP regeneration (20 μg mL^−1^ nLuc).^60,84^ In our study, final yields were increased up to 7.2-fold to 50 μg mL^−1^ for the production of nLuc, exceeding creatine phosphate powered CFPS. Drug treatment contributed to 0.42–39.87% increase in cost of CFPS (Table S2, ESI†). Given the increased productivity of extracts, this translates up to a considerable 9.9-fold increased cost-efficiency from a previous 3.7 μg protein per $ reagent cost to 35.5 μg protein per $ reagent cost using the highly effective nafarelin and cerdulatinib combination (Fig. 6).^60^ Furthermore, drug-induced extract rewiring gave a 44-fold increased yield of the larger Mdm2 (55 kDa) test protein in glucose driven reactions, pointing to even higher cost savings.

In summary, chemoproteomic-assisted phenotypic screening has enabled the enhancement of protein expression yields in yeast and human CFPS. The approach outlined also highlights the utility of CFPS as a surrogate platform to investigate off-target drug activities and toxicity.

## Conclusions

Advances in CFPS technology have principally been guided by rational design and systematic optimization of known user-defined components such as nucleic acid template(s) encoding protein(s) of interest and energy regeneration systems. In this study, we applied a novel target-agnostic phenotypic screening approach to identify/repurpose FDA-approved drugs that improve heterologous protein expression in yeast (*S. cerevisiae)* lysates, one of the least productive CFPS platforms. The top hits increased protein expression up to 44-fold, and yielded the highest levels reported for a model enzyme in *S. cerevisiae* lysates. This approach, coupled with proteomic-enabled target deconvolution, uncovered both novel CFPS agonists and targets, highlighting deleterious background pathways that are now more tractable. The methodology developed aids in the deconvolution of complex biology and complements existing approaches to make CFPS technology both cheaper and more accessible.

## Materials and methods

### Generation of CFPS constructs

pJL1-sfGFP plasmid was purchased from Addgene (#102634), and modified to include 5′UTR and 3′UTR sequences for efficient cap-independent translation in yeast CFPS.^48^ The ribosome binding site downstream of the T7 promoter was replaced with a tobacco mosaic virus 5′UTR fragment of Ω sequence by inverse PCR, retaining an NdeI restriction site. Similarly using inverse PCR, a stretch of 90-nt poly(A) sequence was inserted immediately after the sfGFP stop codon with a BamHI restriction site in between. The sfGFP coding region was subsequently replaced by In-Fusion cloning (Takara) whereby the vector was digested with restriction enzymes NdeI and BamHI (New England BioLabs Inc.) and an insert was generated by standard PCR. All primers were purchased from integrated DNA technologies (IDT). For primer sequences, refer to Supplementary File 1 (ESI†).

### Yeast extract preparation

S30 yeast extract was prepared with reference to established protocols.^48,50^ The S288c yeast strain was a kind gift from A/Prof. Yew Wen Shan from Yong Loo Lin School of Medicine, NUS. BY4743 WT and BY4743 *SSA1*Δ knockout strains were a kind gift from Dr Prakash Arumugam from the Institute of Food and Biotechnology Innovation (SIFBI) in A*STAR. The S288c yeast strain was used for drug screening and subsequent experiments unless stated otherwise. Briefly, yeast was grown in YPD broth (ForMedium™) supplemented with 50 mM Potassium Phosphate (pH 5.5) at 30 °C with shaking (250 rpm) and harvested at OD 10–12, unless specified otherwise. Frozen pellets were resuspended in Lysis Buffer containing 20 mM HEPES-KOH pH 7.4, 100 mM potassium glutamate, 2 mM magnesium glutamate, 2 mM DTT, and 0.5 mM PMSF at 1 mL mg^−1^ of pellet. Cells were lysed by means of high-pressure homogenization using an EmulsiFlex®-C3 high pressure homogenizer (25 000 psi, single pass) and dialyzed against the same lysis buffer. The final yeast extract concentration was determined using a Bradford Assay (Bio-Rad). The aliquots of the extract were flash frozen in liquid nitrogen and stored at −80 °C.

### Cell free protein synthesis (CFPS)

CFPS reactions were set up with respect to optimised protocols.^50^ Briefly, yeast extract was used at 50% (v/v) in a 15 μL reaction mix. The reactions were set in 1.5 mL Eppendorf tubes at 22 °C for 2.5 h for creatine phosphate-driven CFPS and for 6 h for glucose-driven CFPS. T7RNAP was purified according to Brodiazhenko, and added to the reaction mix at 0.027 mg mL^−1^.^44^ pJl1 plasmids with target genes, purified with Maxi kit (Thermo Fisher Scientific), were used as CFPS templates. Reactions were supplemented with 0.08 mM of each of 20 amino acids, 1.5 mM each of ATP, UTP, GTP and CTP, 1.7 mM DTT, 2 mM Putrescine and 0.5 mM Spermidine. The Mg^2+^ and K^+^ concentrations were optimized for each extract and template concentration was optimized for creatine phosphate and glucose energy systems. Creatine phosphate-driven CFPS included 0.27 mg mL^−1^ creatine kinase (Roche), 25 mM creatine phosphate (Roche) and 1.7 mM DTT (Fig. S7, ESI†). Glucose-driven CFPS was set following the established protocol and included 25 mM glucose, 0.3 mM cAMP, 10 mM potassium phosphate and 4 mM DTT.^60^

### Nano-luciferase assay

Nano-luciferase activity was determined using a Nano-Glo® Luciferase Assay (Promega). CFPS reactions were diluted in Nano-Glo® Buffer. Luminescence was read using an EnVision 2104 Multilabel Reader every 20 s for at least 15 min, and the maximum reading was recorded. The absolute nano-luciferase yield was determined by comparing the luminescence reads against the standard curve generated with nano-luciferase recombinant protein purchased from Promega (Nluc-HT Protein) (Fig. S8, ESI†).

### Drug screening

A library of 1443 FDA-approved inhibitors was used for drug screening (Selleck). Drugs (100 μM) were pre-incubated with a yeast extract mixture containing T7RNAP and creatine kinase for 15 min at 30 °C. Reactions were set in PCR tubes in 96-well formats with no-drug and no-template controls included in the last column. No-drug controls had a final DMSO concentration (1%) matched to the test groups. Each plate was tested once, and an average of two luciferase readings were obtained. Drug candidates were determined based on the fold change of the luminescence signal of drug-treated reactions with respect to that of untreated controls. A cut-off value of at least 50% increase was used to select candidates for second round testing following the same method. Finalised drug candidates were further tested in a luciferase counter-screen test to eliminate false-positives whereby drugs (100 μM) were added only after completion of CFPS reactions. The synthesized nano-luciferase was incubated with drugs for 5 min at 30 °C and assayed following the method described earlier.

### Western blot assay for p53 and Mdm2

Equal volumes of CFPS reactions were boiled and resolved by SDS-PAGE. CFPS products were blotted with anti-p53 (DO1, 1:1000) or anti-Mdm2 (2A9, 1:1000) antibodies. Both antibodies were a kind gift from Borek Vojtesek. Western blots from at least two independent experiments were performed and one representative image was selected. Densitometry analysis was performed using Image Lab software (Bio-Rad) to determine the fold increase in yields of drug-treated CFPS reactions with respect to drug-free controls.

### HeLa CFPS

HeLa cell lysate-based CFPS reactions from 1-Step Human Coupled IVT Kit (ThermoFisher Scientific) were set up according to the manufacturer's protocol with the addition of a drug pre-incubation step. Bosutinib and dasatinib monohydrate at 100 μM concentrations were added to the HeLa lysate fraction. After 30 min, accessory proteins, reaction mix and pT7-nLuc template (40 ng μL^−1^) were added. The reaction was run at 30 °C in a 1.5 mL Eppendorf tube for 6 h.

### CFPS time-course experiment

CFPS reactions were set as described earlier. The reactions were aliquoted into multiple individual 1.5 mL Eppendorf tubes in 15 μL fractions. At given time points (0–5 hours for creatine phosphate CFPS and 0–8 hours for glucose CFPS) the entire reaction was snap frozen in liquid nitrogen and kept at −20 °C at a given time point. Upon thawing, 1 μL of reaction mix was sampled out in duplicate for nLuc and ATP analysis. nLuc yield was determined as described earlier. ATP levels were measured using The CellTiter-Glo® 2.0 Assay. CFPS samples were diluted 10 times and mixed with an equal volume of 10% TCA solution. Reactions were spun down at 12 000 g for 10 min to remove precipitated proteins. 25 times diluted supernatant was mixed with an equal volume of CellTiter-Glo® 2.0 reagent, incubated in the dark for 10 min and the luminescence signal was read. The ATP levels were determined by comparing the luminescence signal against the ATP standard curve.

### Drug dose and thermal treatment of yeast extract

150 μg of yeast extract was diluted to 45 μl with dilution buffer of 50 mM HEPES pH 7.5, and 10 mM MgCl_2_ with protease inhibitors. A stock solution of 180 mM bosutinib in DMSO was serially diluted to 100× solution with DMSO and a subsequent dilution to 10× working solution with dilution buffer. 5 μl of 10× working solution was added to 45 μl of yeast extract, resulting in a 1% final DMSO concentration and final drug concentrations: vehicle, 0.01 nM, 0.1 nM, 1 nM, 10 nM, 100 nM, 1 μM, 10 μM, 100 μM and 1 mM. This is repeated over three technical replicates where each replicate was run on a separate TMT-10 set. Lysates were incubated with drug for 3 min at room temperature before performing heat treatment at 55 °C in a PCR thermocycler for 3 min, followed by cooling at 4 °C for 3 min. Samples were kept on ice and centrifuged at 20 000*g* for 30 min at 4 °C. The supernatant containing soluble proteins after thermal treatment was transferred to a new tube with care taken to avoid disrupting pelleted proteins.

### Time course treatment of the yeast extract

30 μg of yeast extract was diluted to 50 μl with dilution buffer of 50 mM HEPES pH 7.5, and 10 mM MgCl_2_ with protease inhibitors. The lysates were incubated in a pre-heated 37 °C water bath for 0, 10, 30, 60 and 120 min. Samples taken out of the water bath were placed on ice for 3 min before centrifugation at 20 000*g* for 20 min at 4 °C. The supernatant containing soluble proteins after thermal treatment was transferred to a new tube with care taken to avoid disrupting pelleted proteins.

### Data processing and statistical analysis of CFPS yields

Bar graph results were obtained from at least 3 independent experiments presenting mean ± SD. Statistical analysis between two groups was performed using two-tailed Student's *t*-test with *p* < 0.05 considered significant. *p < 0.05; **p < 0.01; ***p < 0.005; NS, not significant.

### Sample preparation of thermally treated proteins for mass spectrometry proteomics

Proteins were subjected to acetone precipitation by adding 4× volume of ice-cold acetone, incubated at −20 °C for 12 h, then centrifuged at 14 000*g* for 20 min at 4 °C. The supernatant was removed and the protein pellet was air-dried. The protein pellet was reconstituted in 50 μl of 8 M urea in 50 mM HEPES, pH 7.5. Proteins were reduced, alkylated and digested using a slightly modified version of a previously reported protocol. In brief, TCEP was added to 10 mM and incubated at 25 °C for 30 min. CAA was then added to 55 mM and incubated in the dark for 30 min. TEAB buffer was added to dilute 8 M urea to below 2 M urea, and 1 μg lysC was added with incubation for 4 h at 25 °C. TEAB buffer was then added to dilute urea to below 1 M concentration and 1 μg trypsin was added with incubation for 16 h at 25 °C. Digestion was quenched by the addition of TFA to a final 1% (v/v) concentration. Peptides were then desalted using self-packed Empore C18 stage tips. Stage tips were activated with acetonitrile, equilibrated twice with 0.1% formic acid in water. Samples were loaded on the stage-tip twice and then washed twice with 0.1% formic acid in water. Peptides were eluted with 65% acetonitrile, 0.1% formic acid in water. Eluted peptides were dried by vacuum centrifugation. 5 μg of dried peptides were then resolubilized with 7 μl of 100 mM TEAB pH 8.5. 3 μl of TMT-10 labelling reagent was added and incubated at room temperature for 16 h. Labelling was quenched by the addition of 5% hydroxylamine and incubated for 15 min. The samples were pooled together and diluted with 100 μl ammonium formate pH 10 in water. Desalting at high pH was performed using self-packed spin columns (MoBiTec) fritted with a 10 μm pore size filter and loaded with solid phase, ReproSil Pur Basic resin 10 μm particle size (Dr Maisch), in acetonitrile. Spin columns were then equilibrated with 100% acetonitrile and conditioned by passing 10 mM ammonium formate pH 10 through twice. The samples were loaded and then eluted with 50% acetonitrile in 10 mM ammonium formate pH 10. Fractions were dried by vacuum centrifugation and stored at −20 °C before mass spectrometry analysis.

### Liquid chromatography mass spectrometry proteomics

TMT-10 labelled peptides were resuspended in water with 2% acetonitrile, 0.5% acetic acid and 0.06% trifluoroacetic acid and loaded on a heated (50 °C) Easy-Spray 75 μm × 50 cm column on a Vanquish Neo (Thermo Scientific) liquid chromatography system coupled to an Orbitrap Eclipse Tribrid Mass Spectrometer (Thermo Scientific) with an EASY-Spray source. Peptides were resolved at a flow rate of 300 ml min^−1^, with pre-column equilibration by 100% mobile phase A (0.1% formic acid in water) and resolved by increasing mobile phase B (80% acetonitrile in water with 0.1% formic acid) with a gradient as follows: 0–35% B for 75 min, 35–50% B for 8 min, 50–100% B for 3 min, 100% B for 5 min. The column was equilibrated with 100% A post-run. Mass spectra were collected in Data-Dependent mode with a cycle time of 3 s between master scans. MS1 scans were performed in the Orbitrap with 60 K resolution, AGC target of 400 000 and maximum injection time of 100 ms. MS2 scans collected by Orbitrap with 50 K resolution, 42% HCD collision energy, first mass set at 110, AGC target of 75 000, and maximum injection time of 100 ms.

Mass spectra raw files were searched with SequestHT in Proteome Discoverer 3.0 against a *Saccharomyces cerevisiae* database (retrieved Mar 2017), with the following parameters: precursor mass tolerance of 10 ppm, fragment mass tolerance of 0.06 Da, and trypsin as the enzyme with a maximum of 3 missed cleavages. TMT 10plex was set as the quantification method, with static modification for carbamidomethyl (C) and TMT modification (protein N-terminus, K), and dynamic modifications for acetyl (protein N-terminus), oxidation (M) and deamidation (N,Q). A strict 1% false discovery rate was set using the percolator node, and reporter ions quantifier nodes were added to the workflow for TMT10 quantification. Output search files were exported as separate.txt files for each of the three replicates.

### Data processing and analysis of mass spectrometry data

The three data sets were first individually filtered for missing values and only proteins that were not missing across all samples were retained. The protein abundance at 10 nM for all three replicates and at 1 mM for one of the replicates (set 1) showed systematic shifts in the measurements and were very different from the rest of the samples that cannot be corrected numerically. Thus, we decided to drop these 4 samples from the downstream analysis and used 0.01 nM as the lowest drug concentration value. Next, they were combined into a single data set using 542 common proteins across the three sets. We computed the coefficient-of-variation (%CV) using the lowest three concentrations (0.01 nM, 0.1 nM and 1 nM) and the highest three concentrations (10 μM, 100 μM and 1 mM) for each protein in each replicate. Keeping only proteins with CV below 20% at the highest and lowest three concentrations across all three replicate sets, we retained 457 proteins for the downstream analysis. To remove the differences across the TMT sets, the data was normalized at each concentration against the lowest concentration value to derive the relative protein ratios with respect to 0.01 nM. Then for each replicate, we calculated the mean protein relative ratio at the three highest and three lowest drug concentrations. The values were averaged across replicates and the mean fold change is computed by dividing the average at the higher concentrations by the average at the lower concentrations. Proteins were ranked and prioritized by decreasing magnitude of mean fold changes and those with greater than 1.1 fold increase were subjected to gene ontology (GO) enrichment analysis for biological processes,^89^ and plotted using SRplot.^90^ Protein interactors were plotted using Cytoscape v3.10.0,^91^ using StringApp^92^ to retrieve and map STRING protein interactors. A loess curve was used to fit the changes in the protein abundances across the nine concentrations to model the dose response curves. All analyses were carried out using R studio version 4.2.3.^93^

## Data availability

Raw mass spectrometry spectra and search data were uploaded to the jPost repository^94^ with the following accession numbers: JPST002427 (jPOST) and PXD047986 (ProteomeXchange).

(For reviewers, access key 1584, URL: https://repository.jpostdb.org/preview/11885796746582ad707f418).

## Author contributions

Conceptualization, J. G.; methodology, J. G., Z. L. and R. S.; investigation – Z. L., Z. S., H. K., W. S. and S. C.; writing – original draft, Z. L. and J. G.; writing – review & editing, Z. L., Z. S., H. K., W. S., R. S. and J. G. Funding acquisition, R. S. and J. G.; supervision, R. S. and J. G.

## Conflicts of interest

There are no conflicts to declare.

## Supplementary Material

CB-005-D4CB00004H-s001

CB-005-D4CB00004H-s002

CB-005-D4CB00004H-s003

CB-005-D4CB00004H-s004

CB-005-D4CB00004H-s005

## References

[cit1] Vincent F., Nueda A., Lee J., Schenone M., Prunotto M., Mercola M. (2022). Nat. Rev. Drug Discovery.

[cit2] Lee J. A., Berg E. L. (2013). J. Biomol. Screening.

[cit3] Conway L. P., Li W., Parker C. G. (2021). Cell Chem. Biol..

[cit4] Eder J., Sedrani R., Wiesmann C. (2014). Nat. Rev. Drug Discovery.

[cit5] Carlson E. D., Gan R., Hodgman C. E., Jewett M. C. (2012). Biotechnol. Adv..

[cit6] Gregorio N. E., Levine M. Z., Oza J. P. (2019). Methods Protoc..

[cit7] Silverman A. D., Karim A. S., Jewett M. C. (2020). Nat. Rev. Genet..

[cit8] Dondapati S. K., Stech M., Zemella A., Kubick S. (2020). BioDrugs.

[cit9] Brookwell A., Oza J. P., Caschera F. (2021). Life.

[cit10] Moore S. J., MacDonald J. T., Freemont P. S. (2017). Biochem. Soc. Trans..

[cit11] Tay Y., Ho C., Droge P., Ghadessy F. J. (2010). Nucleic Acids Res..

[cit12] Wei S. J., Joseph T., Sim A. Y., Yurlova L., Zolghadr K., Lane D., Verma C., Ghadessy F. (2013). PLoS One.

[cit13] Goh W., Lane D., Ghadessy F. (2010). Cell Cycle.

[cit14] Tabuchi T., Yokobayashi Y. (2021). RSC Chem. Biol..

[cit15] Maini R., Umemoto S., Suga H. (2016). Curr. Opin. Chem. Biol..

[cit16] Meyer C., Nakamura Y., Rasor B. J., Karim A. S., Jewett M. C., Tan C. (2021). Life.

[cit17] Yin G., Garces E. D., Yang J., Zhang J., Tran C., Steiner A. R., Roos C., Bajad S., Hudak S., Penta K., Zawada J., Pollitt S., Murray C. J. (2012). MAbs.

[cit18] Martin R. W., Majewska N. I., Chen C. X., Albanetti T. E., Jimenez R. B. C., Schmelzer A. E., Jewett M. C., Roy V. (2017). ACS Synth. Biol..

[cit19] Yang J. P., Cirico T., Katzen F., Peterson T. C., Kudlicki W. (2011). BMC Biotechnol..

[cit20] Zemella A., Grossmann S., Sachse R., Sonnabend A., Schaefer M., Kubick S. (2017). Sci. Rep..

[cit21] Wuu J. J., Swartz J. R. (2008). Biochim. Biophys. Acta.

[cit22] Goerke A. R., Swartz J. R. (2009). Biotechnol. Bioeng..

[cit23] Bundy B. C., Franciszkowicz M. J., Swartz J. R. (2008). Biotechnol. Bioeng..

[cit24] Furukawa H., Inaba H., Sasaki Y., Akiyoshi K., Matsuura K. (2022). RSC Chem. Biol..

[cit25] Goto Y., Ito Y., Kato Y., Tsunoda S., Suga H. (2014). Chem. Biol..

[cit26] Kim T.-W., Keum J.-W., Oh I.-S., Choi C.-Y., Kim H.-C., Kim D.-M. (2007). J. Biotechnol..

[cit27] Gan R., Cabezas M. D., Pan M., Zhang H., Hu G., Clark L. G., Jewett M. C., Nicol R. (2022). ACS Synth. Biol..

[cit28] Jewett M. C., Swartz J. R. (2004). Biotechnol. Bioeng..

[cit29] Burrington L. R., Watts K. R., Oza J. P. (2021). ACS Synth. Biol..

[cit30] Choudhury A., Hodgman C. E., Anderson M. J., Jewett M. C. (2014). Biochem. Eng. J..

[cit31] Hong S. H., Kwon Y.-C., Martin R. W., Des Soye B. J., de Paz A. M., Swonger K. N., Ntai I., Kelleher N. L., Jewett M. C. (2015). ChemBioChem.

[cit32] Contreras-Llano L. E., Meyer C., Liu Y., Sarker M., Lim S., Longo M. L., Tan C. (2020). Nat. Commun..

[cit33] Calhoun K. A., Swartz J. R. (2005). Biotechnol. Bioeng..

[cit34] Wang P. H., Fujishima K., Berhanu S., Kuruma Y., Jia T. Z., Khusnutdinova A. N., Yakunin A. F., McGlynn S. E. (2020). ACS Synth. Biol..

[cit35] Jaroentomeechai T., Stark J. C., Natarajan A., Glasscock C. J., Yates L. E., Hsu K. J., Mrksich M., Jewett M. C., DeLisa M. P. (2018). Nat. Commun..

[cit36] Sachse R., Dondapati S. K., Fenz S. F., Schmidt T., Kubick S. (2014). FEBS Lett..

[cit37] Jin X., Kightlinger W., Hong S. H. (2019). Methods Protoc..

[cit38] Martin R. W., Des Soye B. J., Kwon Y.-C., Kay J., Davis R. G., Thomas P. M., Majewska N. I., Chen C. X., Marcum R. D., Weiss M. G., Stoddart A. E., Amiram M., Ranji Charna A. K., Patel J. R., Isaacs F. J., Kelleher N. L., Hong S. H., Jewett M. C. (2018). Nat. Commun..

[cit39] Caschera F., Noireaux V. (2014). Biochimie.

[cit40] Guarino C., DeLisa M. P. (2012). Glycobiology.

[cit41] Jiang X., Ookubo Y., Fujii I., Nakano H., Yamane T. (2002). FEBS Lett..

[cit42] Panthu B., Ohlmann T., Perrier J., Schlattner U., Jalinot P., Elena-Herrmann B., Rautureau G. J. P. (2018). ACS Synth. Biol..

[cit43] Aleksashin N. A., Chang S. T., Cate J. H. D. (2023). RNA.

[cit44] Brodiazhenko T., Johansson M. J. O., Takada H., Nissan T., Hauryliuk V., Murina V. (2018). Front. Microbiol..

[cit45] Mattanovich D., Sauer M., Gasser B. (2014). Microb. Cell Fact..

[cit46] Nielsen J. (2013). Bioengineered.

[cit47] Wang X., Liu J., Zheng Y., Li J., Wang H., Zhou Y., Qi M., Yu H., Tang W., Zhao W. M. (2008). J. Biosci. Bioeng..

[cit48] Gan R., Jewett M. C. (2014). Biotechnol. J..

[cit49] Martinez Molina D., Jafari R., Ignatushchenko M., Seki T., Larsson E. A., Dan C., Sreekumar L., Cao Y., Nordlund P. (2013). Science.

[cit50] Hodgman C. E., Jewett M. C. (2013). Biotechnol. Bioeng..

[cit51] Ricciuti B., Baglivo S., De Giglio A., Chiari R. (2018). Ther. Adv. Respir. Dis..

[cit52] Remsing Rix L. L., Rix U., Colinge J., Hantschel O., Bennett K. L., Stranzl T., Muller A., Baumgartner C., Valent P., Augustin M., Till J. H., Superti-Furga G. (2009). Leukemia.

[cit53] Coffey G., Betz A., DeGuzman F., Pak Y., Inagaki M., Baker D. C., Hollenbach S. J., Pandey A., Sinha U. (2014). J. Pharmacol. Exp. Ther..

[cit54] Wissner A., Mansour T. S. (2008). Arch. Pharm..

[cit55] Aguilera D. G., Tsimberidou A. M. (2009). Ther. Clin. Risk Manage..

[cit56] Letassy N. A., Thompson D. F., Britton M. L., Suda, Sr. R. R. (1990). DICP, Ann. Pharmacother..

[cit57] Mohapatra S. S., Dwibedy S. K., Padhy I. (2021). J. Biosci..

[cit58] Islam M. R., Ihenacho K., Park J. W., Islam I. S. (2019). Sci. Rep..

[cit59] Chamberlin M., Ring J. (1973). J. Biol. Chem..

[cit60] Anderson M. J., Stark J. C., Hodgman C. E., Jewett M. C. (2015). FEBS Lett..

[cit61] Dai L., Zhao T., Bisteau X., Sun W., Prabhu N., Lim Y. T., Sobota R. M., Kaldis P., Nordlund P. (2018). Cell.

[cit62] Franken H., Mathieson T., Childs D., Sweetman G. M., Werner T., Togel I., Doce C., Gade S., Bantscheff M., Drewes G., Reinhard F. B., Huber W., Savitski M. M. (2015). Nat. Protoc..

[cit63] Reinhard F. B., Eberhard D., Werner T., Franken H., Childs D., Doce C., Savitski M. F., Huber W., Bantscheff M., Savitski M. M., Drewes G. (2015). Nat. Methods.

[cit64] Savitski M. M., Reinhard F. B., Franken H., Werner T., Savitski M. F., Eberhard D., Martinez Molina D., Jafari R., Dovega R. B., Klaeger S., Kuster B., Nordlund P., Bantscheff M., Drewes G. (2014). Science.

[cit65] Almqvist H., Axelsson H., Jafari R., Dan C., Mateus A., Haraldsson M., Larsson A., Martinez Molina D., Artursson P., Lundback T., Nordlund P. (2016). Nat. Commun..

[cit66] Liang Y. Y., Bacanu S., Sreekumar L., Ramos A. D., Dai L., Michaelis M., Cinatl J., Seki T., Cao Y., Coffill C. R., Lane D. P., Prabhu N., Nordlund P. (2022). Cell Chem. Biol..

[cit67] Lu Q., Zhang Y., Hellner J., Giannini C., Xu X., Pauwels J., Ma Q., Dejonghe W., Han H., Van de Cotte B., Impens F., Gevaert K., De Smet I., Friml J., Molina D. M., Russinova E. (2022). Proc. Natl. Acad. Sci. U. S. A..

[cit68] Mateus A., Bobonis J., Kurzawa N., Stein F., Helm D., Hevler J., Typas A., Savitski M. M. (2018). Mol. Syst. Biol..

[cit69] Craig E. A., Jacobsen K. (1984). Cell.

[cit70] Contreras-Llano L. E., Tan C. (2018). Synth. Biol..

[cit71] Chappell J., Jensen K., Freemont P. S. (2013). Nucleic Acids Res..

[cit72] Tamaki F., Fisher F., Milne R., Terán F. S.-R., Wiedemar N., Wrobel K., Edwards D., Baumann H., Gilbert I. H., Baragana B., Baum J., Wyllie S. (2022). Antimicrob. Agents Chemother..

[cit73] Hurst G. B., Asano K. G., Doktycz C. J., Consoli E. J., Doktycz W. L., Foster C. M., Morrell-Falvey J. L., Standaert R. F., Doktycz M. J. (2017). Anal. Chem..

[cit74] Rasor B. J., Chirania P., Rybnicky G. A., Giannone R. J., Engle N. L., Tschaplinski T. J., Karim A. S., Hettich R. L., Jewett M. C. (2023). ACS Synth. Biol..

[cit75] Foshag D., Henrich E., Hiller E., Schafer M., Kerger C., Burger-Kentischer A., Diaz-Moreno I., Garcia-Maurino S. M., Dotsch V., Rupp S., Bernhard F. (2018). N Biotechnol..

[cit76] Sharma D., Masison D. C. (2009). Protein Pept. Lett..

[cit77] Werner-Washburne M., Becker J., Kosic-Smithers J., Craig E. A. (1989). J. Bacteriol..

[cit78] Lotz S. K., Knighton L. E., Nitika, Jones G. W., Truman A. W. (2019). Curr. Genet..

[cit79] Kirmizibayrak P. B., Ilhan R., Yilmaz S., Gunal S., Tepedelen B. E. (2018). J. Biochem..

[cit80] Leeming M. G., O'Callaghan S., Licata L., Iannuccelli M., Lo Surdo P., Micarelli E., Ang C. S., Nie S., Varshney S., Ameen S., Cheng H. C., Williamson N. A. (2021). Bioinformatics.

[cit81] Garcia D. C., Dinglasan J. L. N., Shrestha H., Abraham P. E., Hettich R. L., Doktycz M. J. (2021). Metab. Eng. Commun..

[cit82] Zemella A., Thoring L., Hoffmeister C., Kubick S. (2015). ChemBioChem.

[cit83] Vieira Gomes A. M., Souza Carmo T., Silva Carvalho L., Mendonça Bahia F., Parachin N. S. (2018). Microorganisms.

[cit84] Schoborg J. A., Clark L. G., Choudhury A., Hodgman C. E., Jewett M. C. (2016). Synth. Syst. Biotechnol..

[cit85] Zhang L., Liu W. Q., Li J. (2020). Front. Bioeng. Biotechnol..

[cit86] Wang Y., Wang T., Chen X., Lu Y. (2023). Bioresour. Bioprocess.

[cit87] Schoborg J. A., Hodgman C. E., Anderson M. J., Jewett M. C. (2014). Biotechnol. J..

[cit88] Guzman-Chavez F., Arce A., Adhikari A., Vadhin S., Pedroza-Garcia J. A., Gandini C., Ajioka J. W., Molloy J., Sanchez-Nieto S., Varner J. D., Federici F., Haseloff J. (2022). ACS Synth. Biol..

[cit89] Thomas P. D., Ebert D., Muruganujan A., Mushayahama T., Albou L. P., Mi H. (2022). Protein Sci..

[cit90] Tang D., Chen M., Huang X., Zhang G., Zeng L., Zhang G., Wu S., Wang Y. (2023). PLoS One.

[cit91] Shannon P., Markiel A., Ozier O., Baliga N. S., Wang J. T., Ramage D., Amin N., Schwikowski B., Ideker T. (2003). Genome Res..

[cit92] Doncheva N. T., Morris J. H., Gorodkin J., Jensen L. J. (2019). J. Proteome Res..

[cit93] R. C. Team, Journal, 2023

[cit94] Okuda S., Watanabe Y., Moriya Y., Kawano S., Yamamoto T., Matsumoto M., Takami T., Kobayashi D., Araki N., Yoshizawa A. C., Tabata T., Sugiyama N., Goto S., Ishihama Y. (2017). Nucleic Acids Res..

