# Supplementary material for: Engineering cell-free systems by chemoproteomic-assisted phenotypic screening
Source: RSC Chem Biol. 2024 Mar 6;5(4):372–85. doi: 10.1039/d4cb00004h (PMC10989505; doi:10.1039/d4cb00004h)
Supplement: CB-005-D4CB00004H-s001 [file CB-005-D4CB00004H-s001.pdf]

Electronic Supplementary Material (ESI) for RSC Chemical Biology.
This journal is © The Royal Society of Chemistry 2024

Supplementary Figures

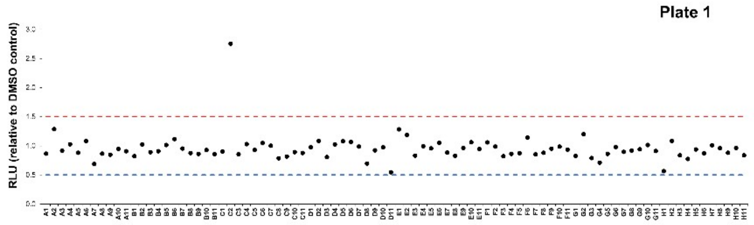

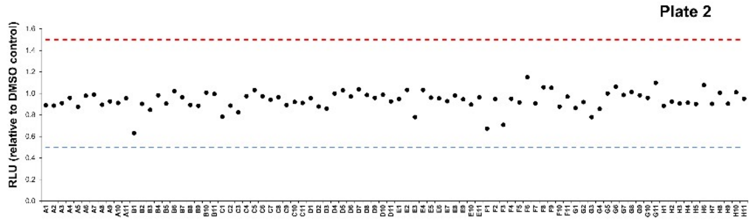

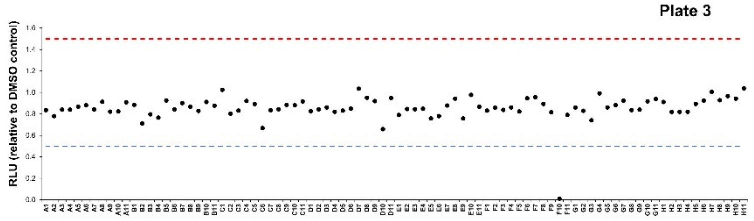

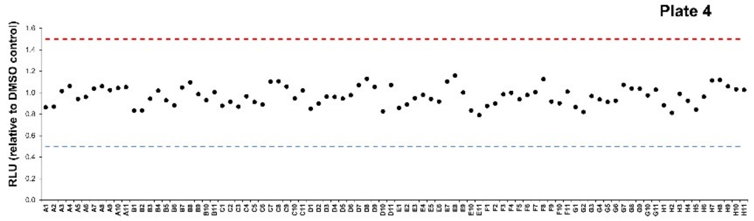

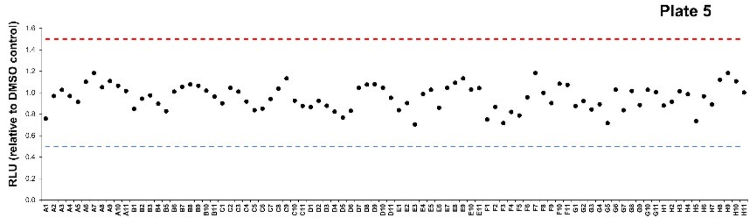

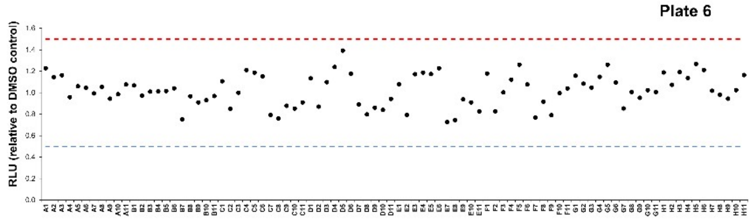

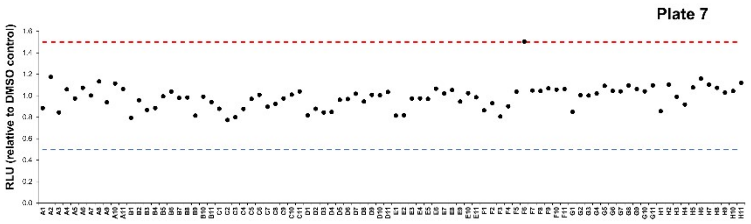
Plate 7
RLU (relative to DMSO control)

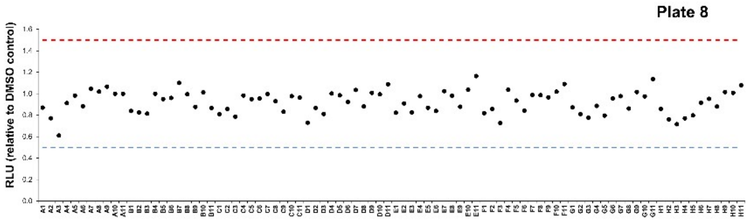
Plate 8
RLU (relative to DMSO control)

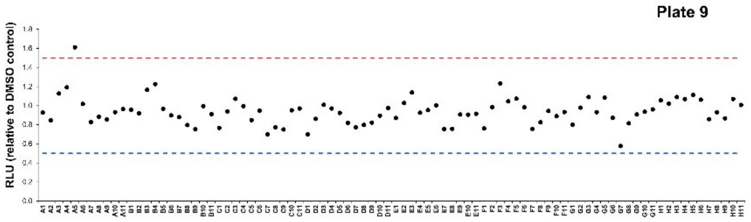
Plate 9
RLU (relative to DMSO control)

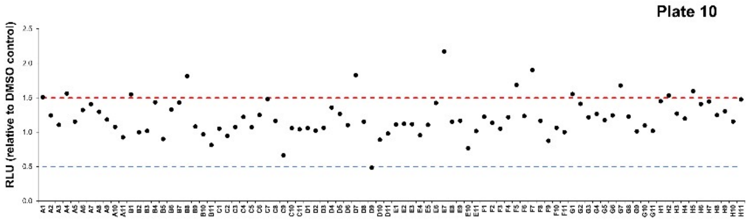
Plate 10
RLU (relative to DMSO control)

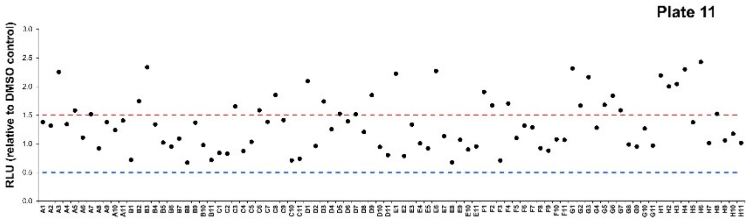
Plate 11
RLU (relative to DMSO control)

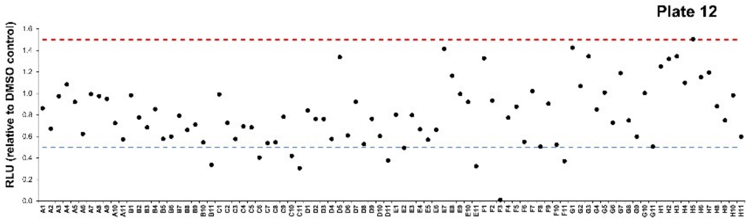
Plate 12
RLU (relative to DMSO control)

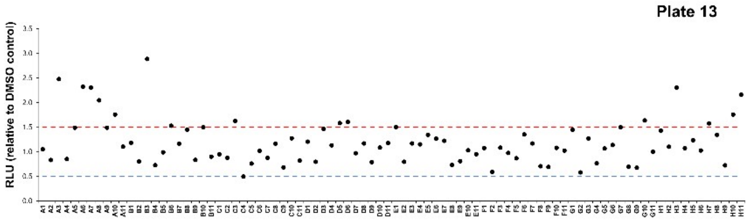
Plate 13
RLU (relative to DMSO control)

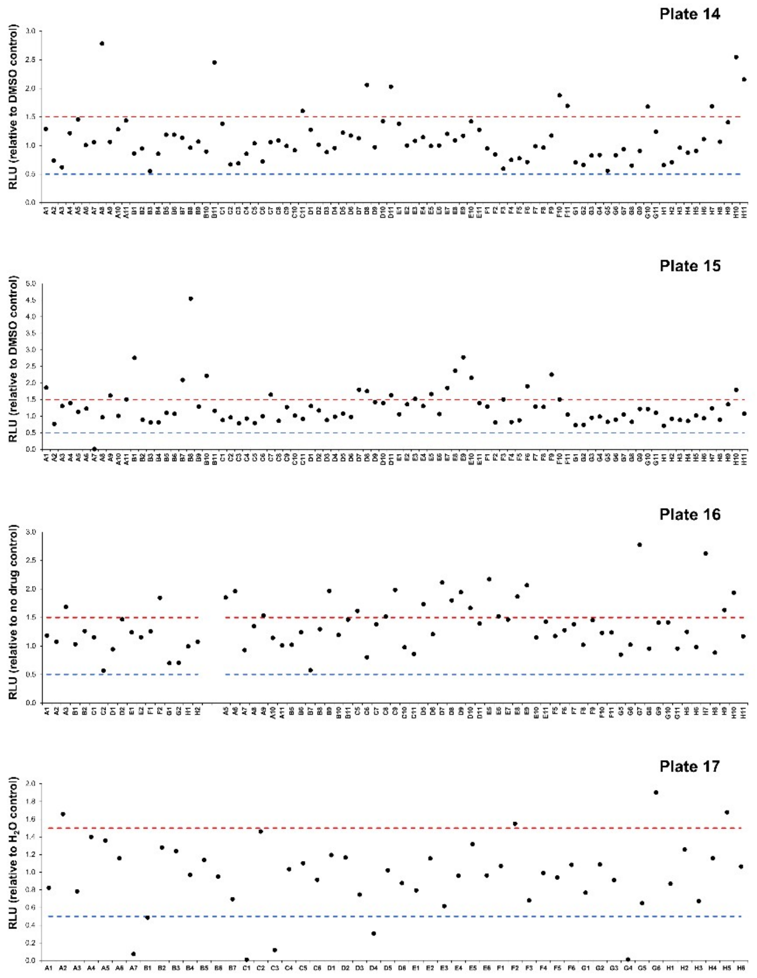

Figure S1. Drug screen with collection of 1430 FDA-approved small molecule agonists

CFPS reactions were screened in 96-well format taking average RLU from two reads to assay nano-luciferase production. CFPS yield was normalised against the average of at least three no-drug control groups to determine fold change. Dashed lines indicate 50% threshold for identification of drug hits that increase (red line) or decrease (blue line) CFPS activity. (Second half of Plate 16 and entire Plate 17 drugs were dissolved in water, while the rest contained 10% DMSO).

A

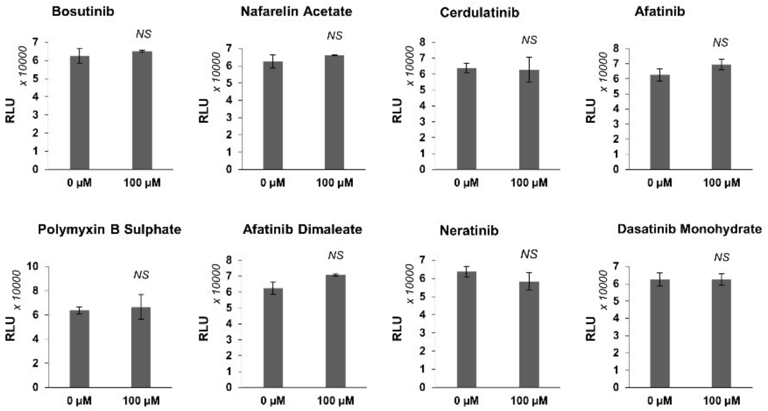

B

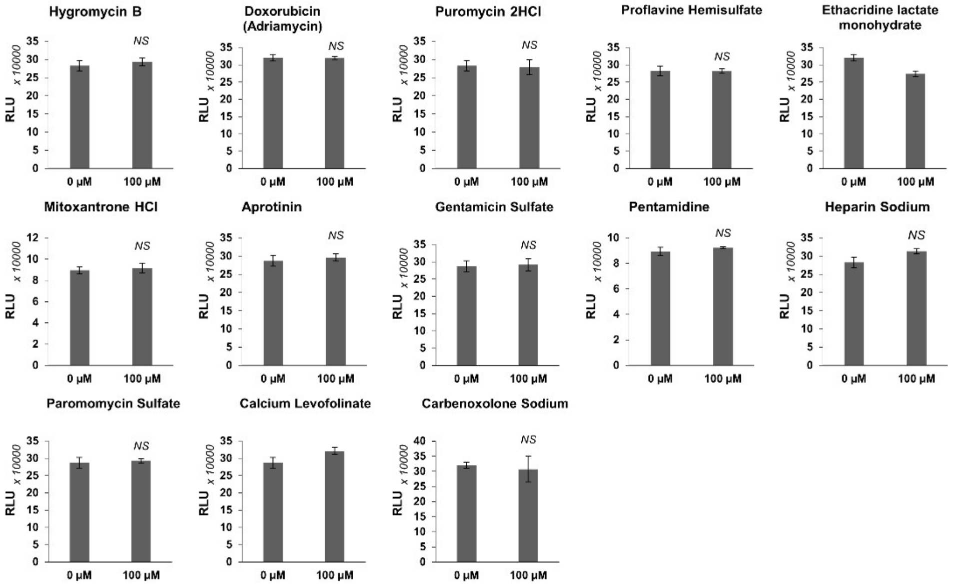

Figure S2. Counter-screen assay.

Effect of identified positive hits (A) and inhibitors (B) on nLuc activity was assessed by adding compounds into CFPS reactions upon completion of nLuc synthesis. n=3 ± SD.

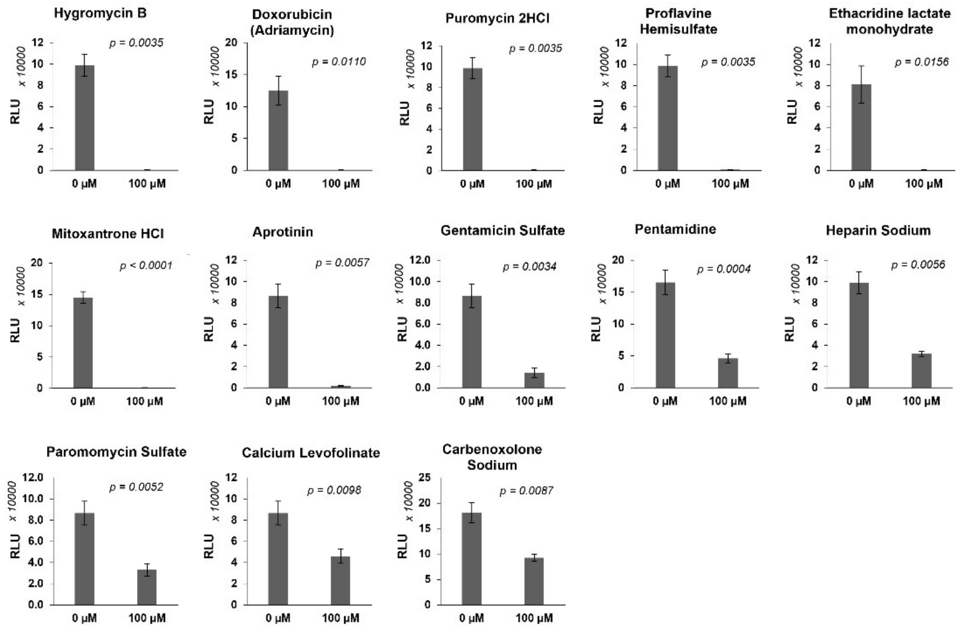

Figure S3. CFPS inhibitors identified in screen.

Selected drug candidates were validated in the second-round screen. Positive hits (Figure 1B) and inhibitors with significant fold change reduction in CFPS yields are shown. n=3 ± SD.

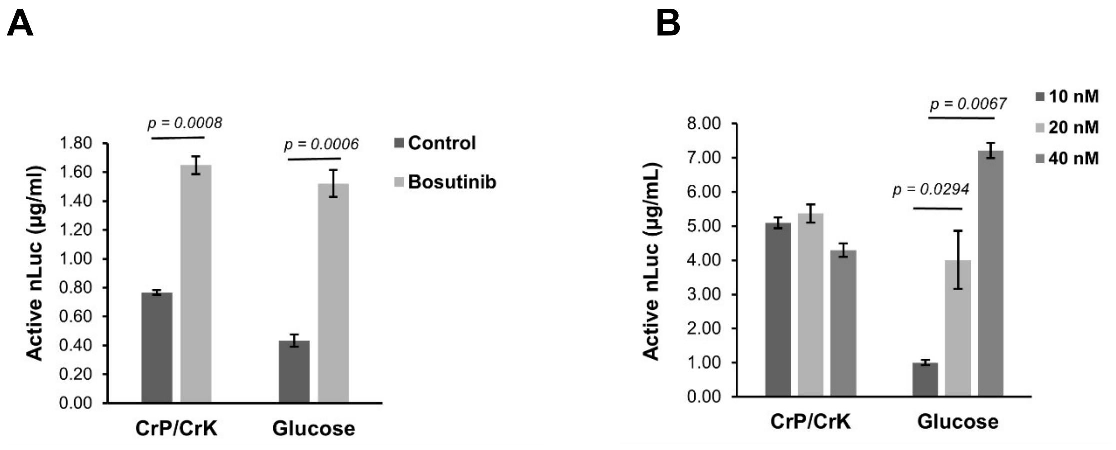

Figure S4. Effect of bosutinib on glucose-driven CFPS reactions

(A) CFPS reactions were set using either creatine phosphate or glucose as secondary energy source. Reactions were treated with 100 µM bosutinib to assess drug effect on nLuc yield. n=3 ± SD.

(B) Creatine phosphate and glucose-driven CFPS reactions were tested with indicated amounts of nLuc plasmid template (10 - 40 nM). n=3 ± SD.

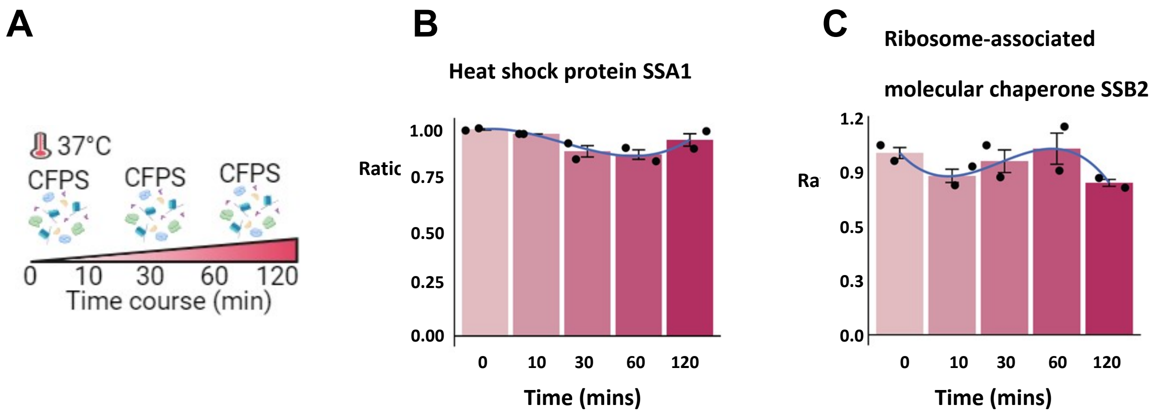

Figure S5. Protein levels of SSA1 and SSB2 remain stable over the course of a CFPS reaction.

(A) Proteomics profiling of CFPS over the course of a reaction at different time points: 0, 10, 30, 60, 120min. Proteins were then subjected to sample processing, TMT labelling and LC-MS acquisition.
(B,C) Protein abundance of SSA1 and SSB2 remain stable over the course of a CFPS reaction.

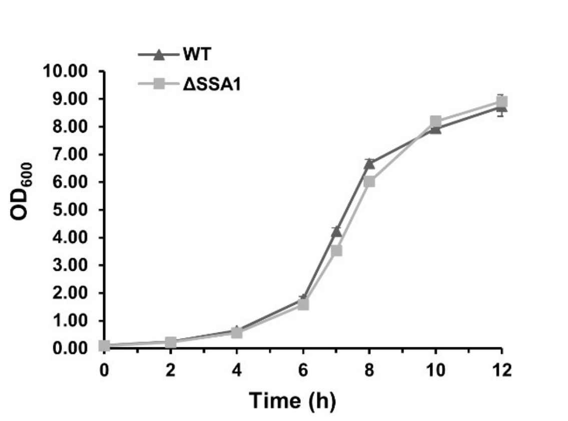

Figure S6. BY4743-WT and BY4743-ΔSSA1 growth curves.

Growth of BY4743-WT and BY4743-Δ*SSA1* was monitored for 12 h from starting OD_{600} = 0.1. n=2 ± SD.

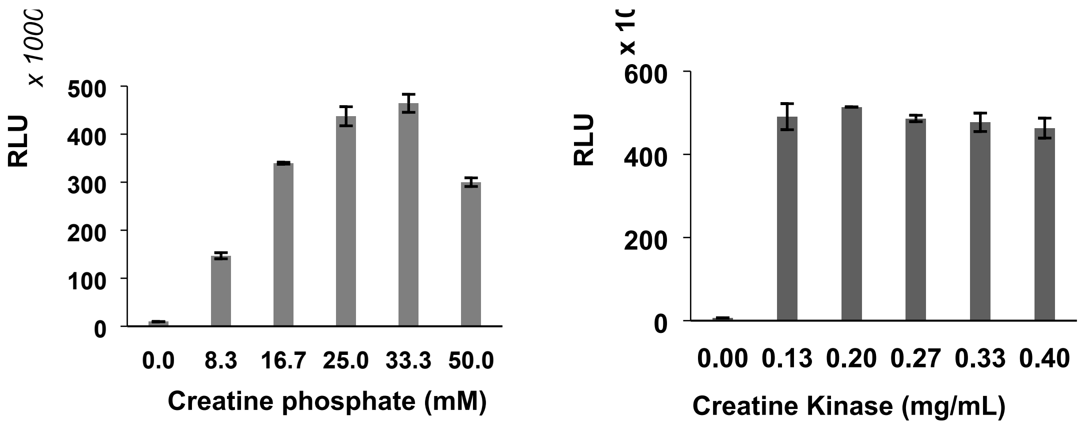

Figure S7. Optimisation of energy regeneration components.

CFPS reactions were set up with varying concentrations of creatine phosphate (0-50 mM) and creatine kinase (0-0.4 mg/mL) to identify optimal conditions. n=3 ± SD.

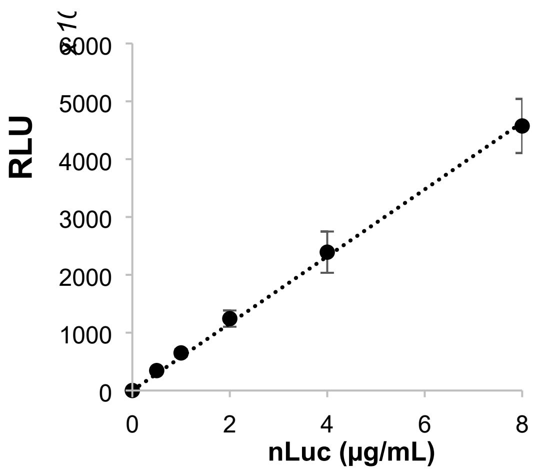

Figure S8. Standard curve of nLuc activity.

Recombinant nLuc protein (Promega) was diluted in commercial buffer from Nano-Glo® Luciferase Assay kit (0-8 µg/mL) and RLU was measured. n=3 ± SD.
