# Supplementary material for: Engineering cell-free systems by chemoproteomic-assisted phenotypic screening
Source: RSC Chem Biol. 2024 Mar 6;5(4):372–85. doi: 10.1039/d4cb00004h (PMC10989505; doi:10.1039/d4cb00004h)
Supplement: CB-005-D4CB00004H-s003 [file CB-005-D4CB00004H-s003.pdf]

Electronic Supplementary Material (ESI) for RSC Chemical Biology.
This journal is © The Royal Society of Chemistry 2024

Table S1 List of drug hits decreasing yeast extract productivity

Drug	Mode of action	Targets	
**Hygromycin B**	Antibiotic<br>inhibits protein synthesis	Ribosomes	30S rRNA
		Ribosome-dependent ATPase	RbbA
**Doxorubicin (Adriamycin)**	Antibiotic<br>induces DNA damage and apoptosis	DNA Topoisomerase	TOP1, TOP2A, TOP2B
		Nucleic acids	DNA
		Nucleolar phosphoprotein	NOLC1
**Puromycin 2HCl**	Antibiotic<br>inhibits protein synthesis	Ribosomal proteins	RPL10L, RPL13A, RPL23, RPL15, RPL19, RPL23A, RSL24D1, RPL26L1, RPL8. RPL37, RPL3, RPL11,
		tRNA-protein transferase	aat
**Proflavine Hemisulfate**	Antiseptic<br>intercalates DNA	Nucleic acids	DNA
**Ethacridine lactate monohydrate**	Antiseptic<br>intercalates DNA	Nucleic acids	DNA
**Mitoxantrone HCl**	type II topoisomerase inhibitor<br>intercalates DNA	DNA Topoisomerase	TOP2A
		Nucleic acids	DNA
**Aprotinin**	Serine protease inhibitor	Serine protease	PRSS1, CTRB1, PLG, KLK1
**Gentamicin Sulfate**	Antibiotic<br>inhibits protein synthesis	Ribosomes	rpsL, 16S rRNA, 23S rRNA,
		Membrane	
		Synthetase	nadE
		Reductase	DHFR
**Pentamidine**	Antiprotozoal agent	PRL Phosphatases	PTP1B

	inhibits synthesis of DNA, RNA, phospholipids and proteins	tRNA-methyltransferase	TRDMT1
		Nucleic acids	DNA
**Heparin sodium**	Anticoagulant<br>Competitive inhibitor of RNA synthesis	Serpin protease inhibitor	SERPINC1
		Coagulation factor X	F10
		Selectin	SELP
		Tyrosine kinase	FGFR1, FGFR2, FGFR4
		Fibroblast growth factor	FGF1, FGF2, FGF4, FGF19
		Cytokine	PF4
		Polymerases	T7RNAP, polA
**Paromomycin Sulfate**	Antibiotic<br>inhibits protein synthesis	Ribosomes	16S rRNA, rpsJ, RPSA, RPL10L
**Calcium Levofolinate**	Calcium salt of folinic acid and analogue of tetrahydrofolate	Serine hydroxymethyltransferase	glyA
**Carbenoxolone Sodium**	Synthetic derivative of glycyrrhetinic acid	Pannexon membrane channels and related innexon channels	11β-HSD
