# Supplementary material for: Engineering cell-free systems by chemoproteomic-assisted phenotypic screening
Source: RSC Chem Biol. 2024 Mar 6;5(4):372–85. doi: 10.1039/d4cb00004h (PMC10989505; doi:10.1039/d4cb00004h)
Supplement: CB-005-D4CB00004H-s004 [file CB-005-D4CB00004H-s004.pdf]

Electronic Supplementary Material (ESI) for RSC Chemical Biology.
This journal is © The Royal Society of Chemistry 2024

Table S2 Cost calculation of drug treated CFPS reactions

Drug (TargetMol [a])	Cost (USD/mg)	Cost CFPS (USD/ L reaction)	% Cost (CrP/CrK)	% Cost (glucose)	nLuc Yield (µg/mL)	Cost efficiency (µg nLuc/USD)	Fold increase in cost efficiency [b]	Fold increase in cost efficiency [c]
Bosutinib	0.88	46.68	1.56%	4.67%	18.1	17.29	4.80	2.51
Cerdulatinib	2.16	96.23	3.21%	9.62%	12.9	11.77	3.27	1.71
Nafarelin Acetate	2.16	302.52	10.08%	30.25%	25.3	19.42	5.40	2.82
Dasatinib	0.20	58.56	1.95%	5.86%	11.5	10.86	3.02	1.57
Neratinib	0.66	14.71	0.49%	1.47%	*N.A.*	*N.A.*	*N.A.*	*N.A.*
Afatinib	0.63	30.61	1.02%	3.06%	*N.A.*	*N.A.*	*N.A.*	*N.A.*
Polymyxin B	0.03	4.16	0.14%	0.42%	*N.A.*	*N.A.*	*N.A.*	*N.A.*
Nafarelin + Cerdulatinib	4.32	398.75	13.29%	39.87%	49.7	35.53	9.87	5.15
Control (current)	0.00	0.00	*N.A.*	*N.A.*	6.9	6.90	1.92	1.00
Control (reported)	0.00	0.00	*N.A.*	*N.A.*	3.6	3.60	1.00	0.52

[a] Cost of drugs obtained from TargetMol website
[b] Fold increase in cost efficiency is calculated with respect to reported cost efficiency using optimised glucose system[1]
[c] Fold increase in cost efficiency is calculated with respect to cost efficiency of untreated control in the current study

Reference:

1. M. J. Anderson, J. C. Stark, C. E. Hodgman and M. C. Jewett, *FEBS Lett*, 2015, **589**, 1723-1727.
