# Supplementary material for: Engineering cell-free systems by chemoproteomic-assisted phenotypic screening
Source: RSC Chem Biol. 2024 Mar 6;5(4):372–85. doi: 10.1039/d4cb00004h (PMC10989505; doi:10.1039/d4cb00004h)

Electronic Supplementary Material (ESI) for RSC Chemical Biology.
This journal is © The Royal Society of Chemistry 2024

Fig.2D Drug effect on p53 synthesis (trial 1)

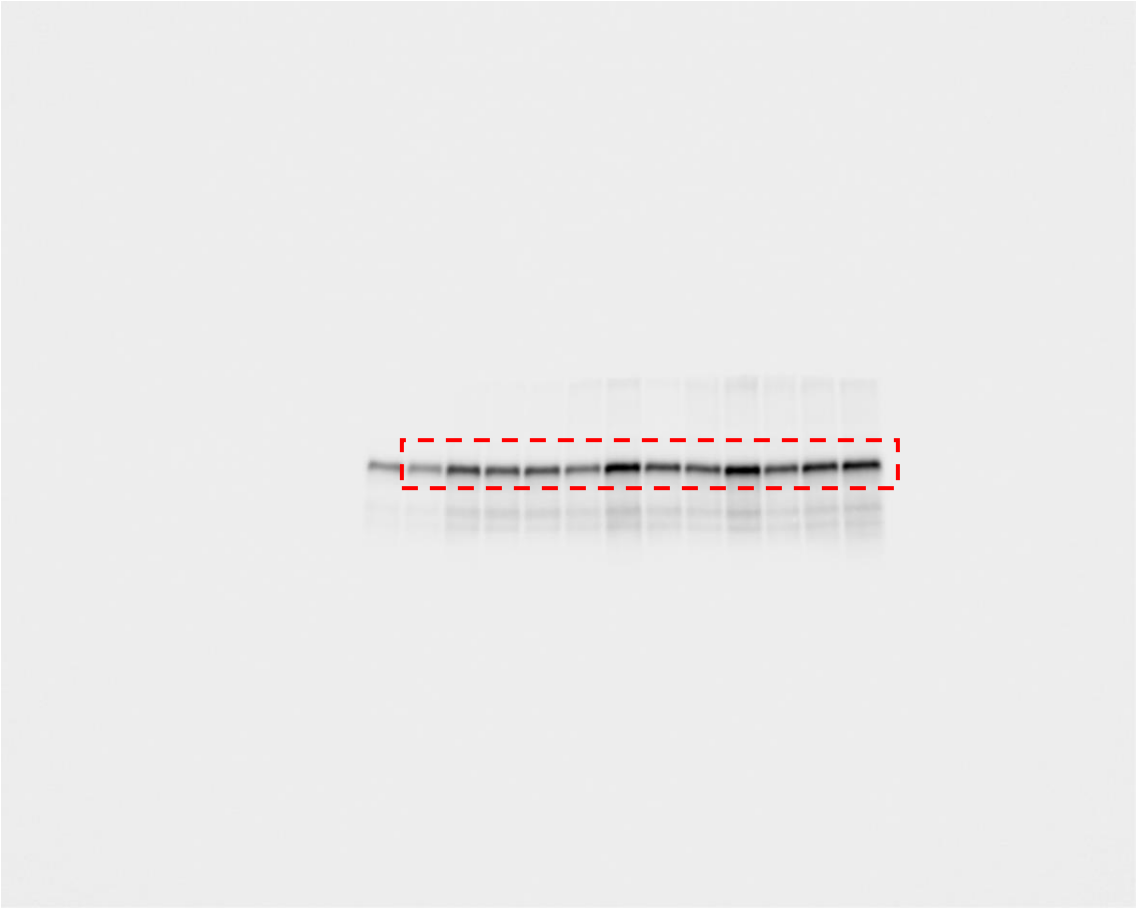

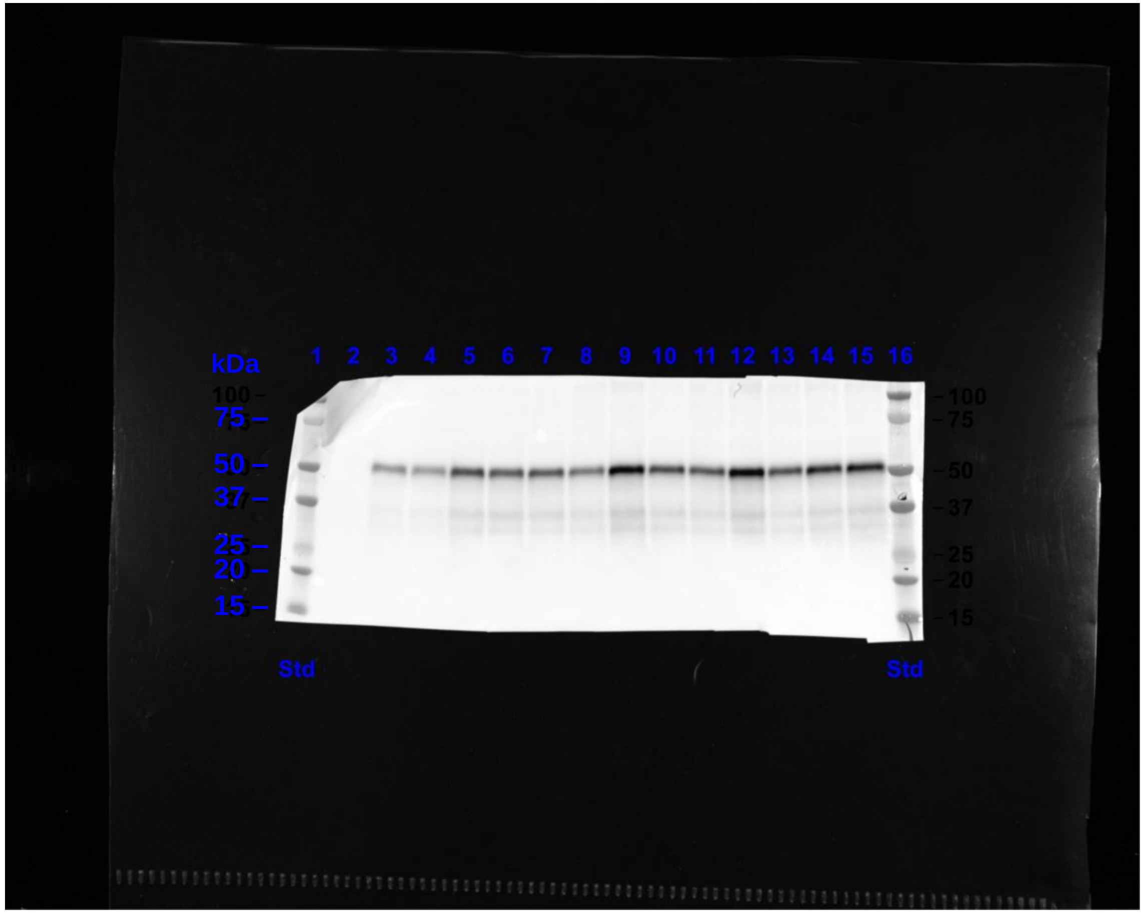

Fig.2D Drug effect on p53 synthesis (trial 2)

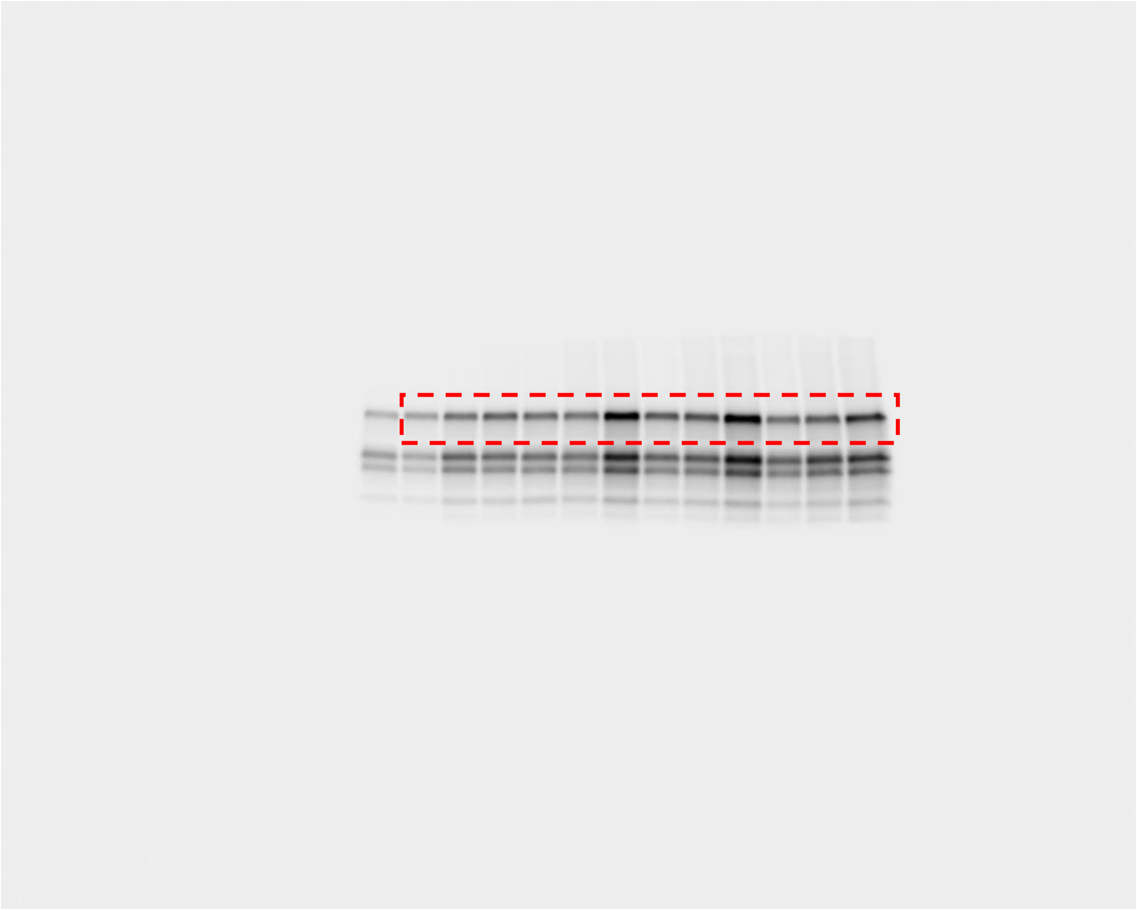

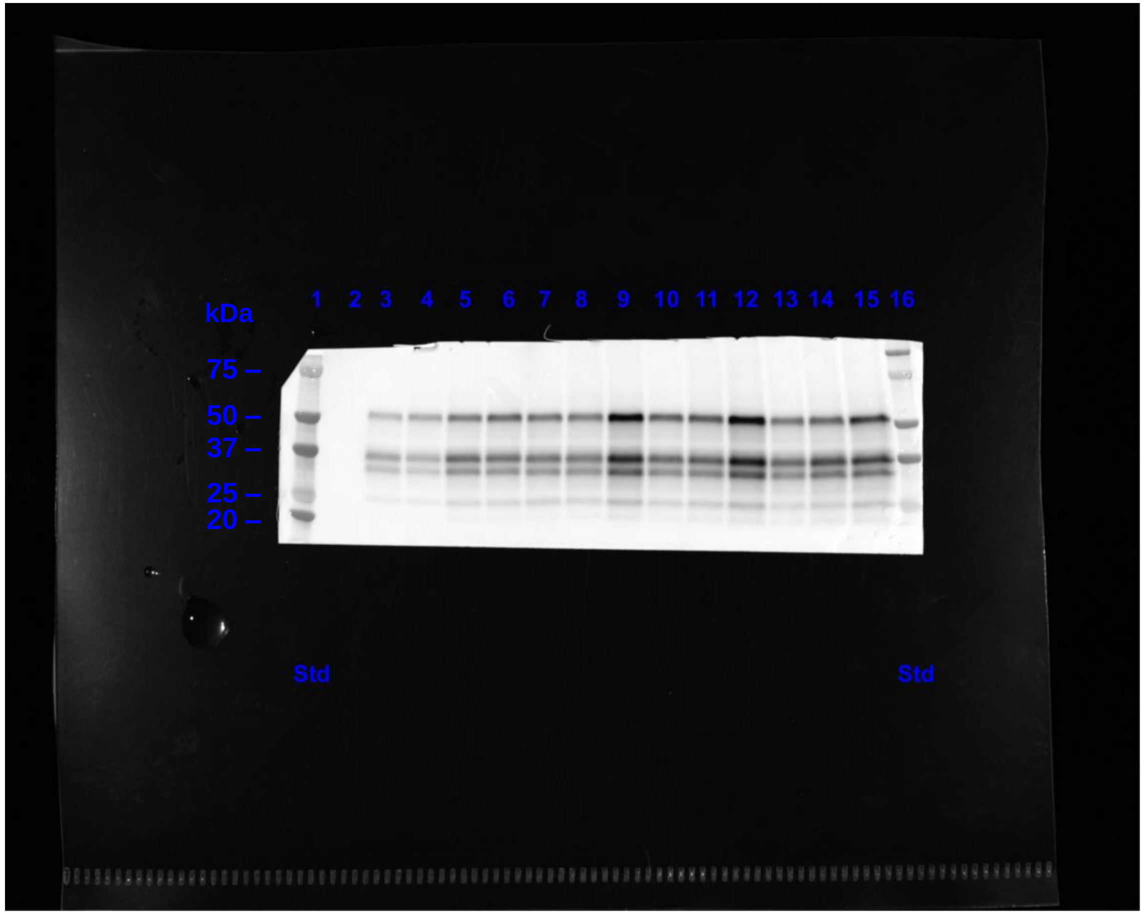

Fig.2D Drug effect on p53 synthesis (trial 3)

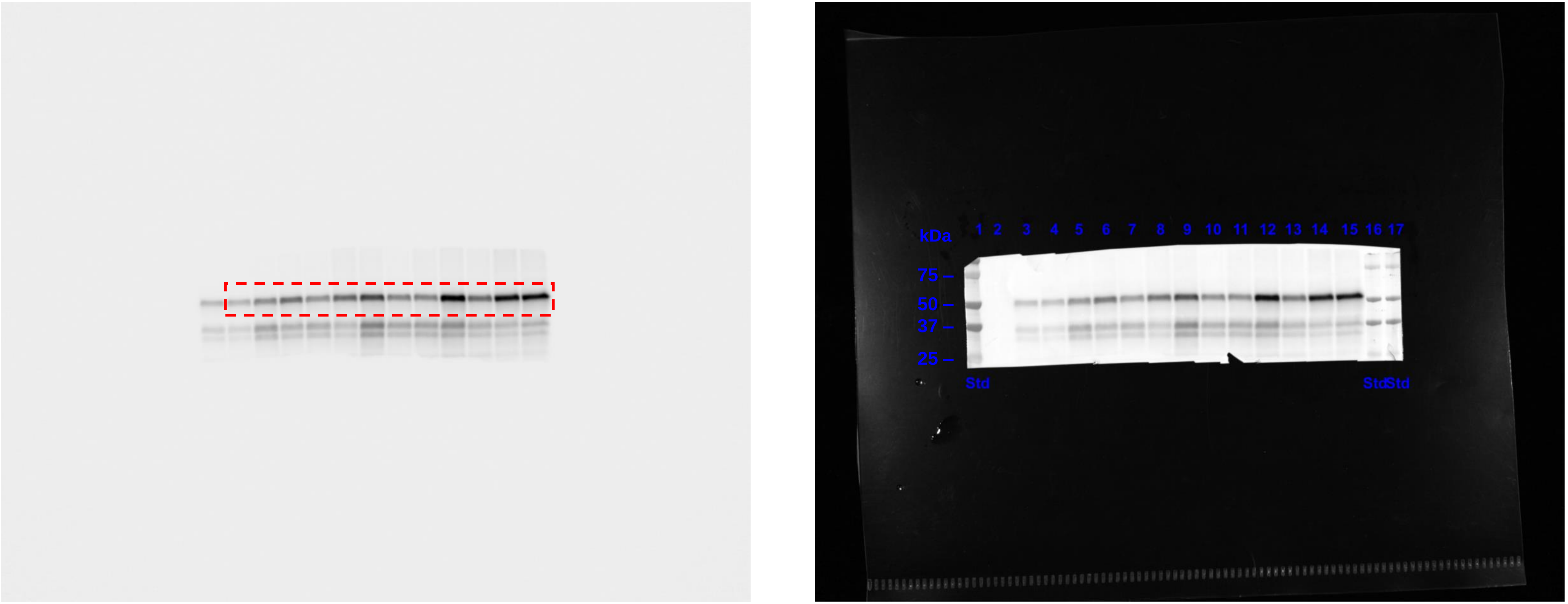

Fig.6C Mdm2 synthesis with WT and ΔSSA1 extracts (trial 1)

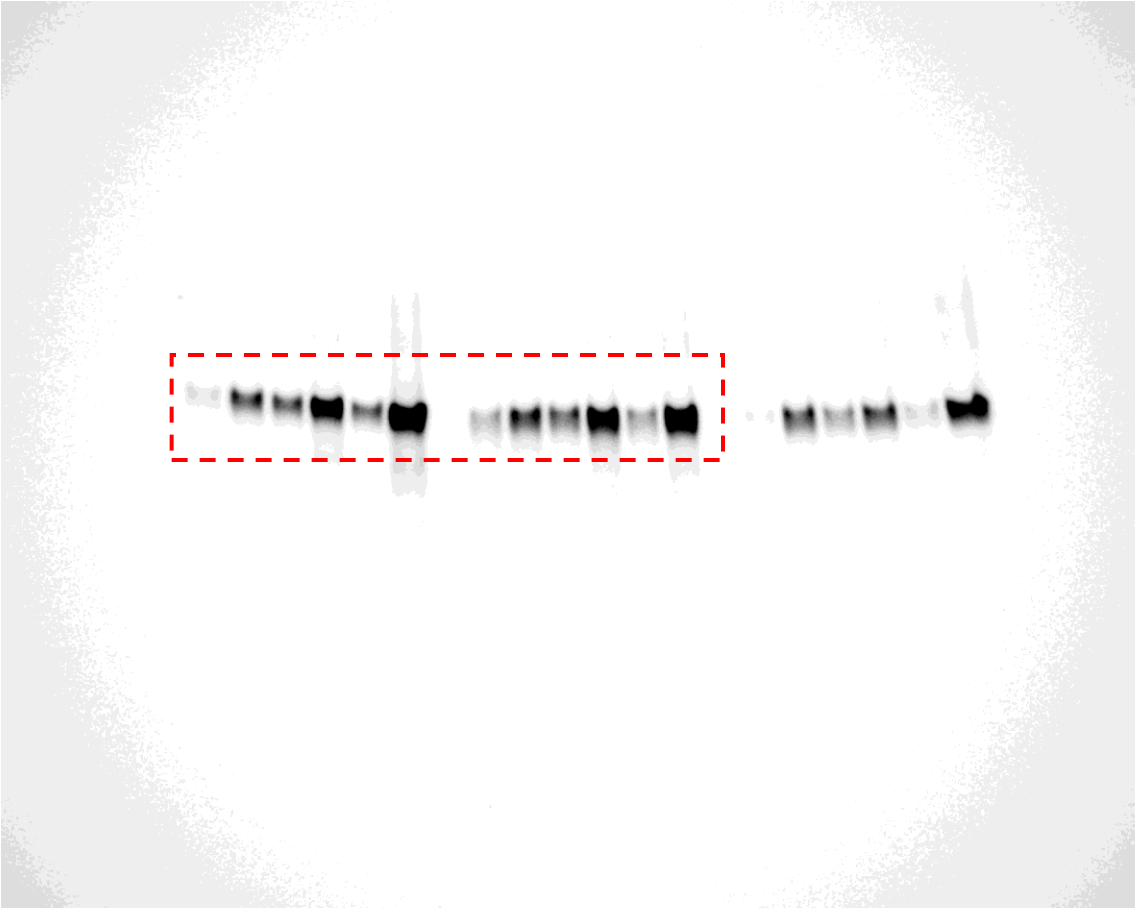

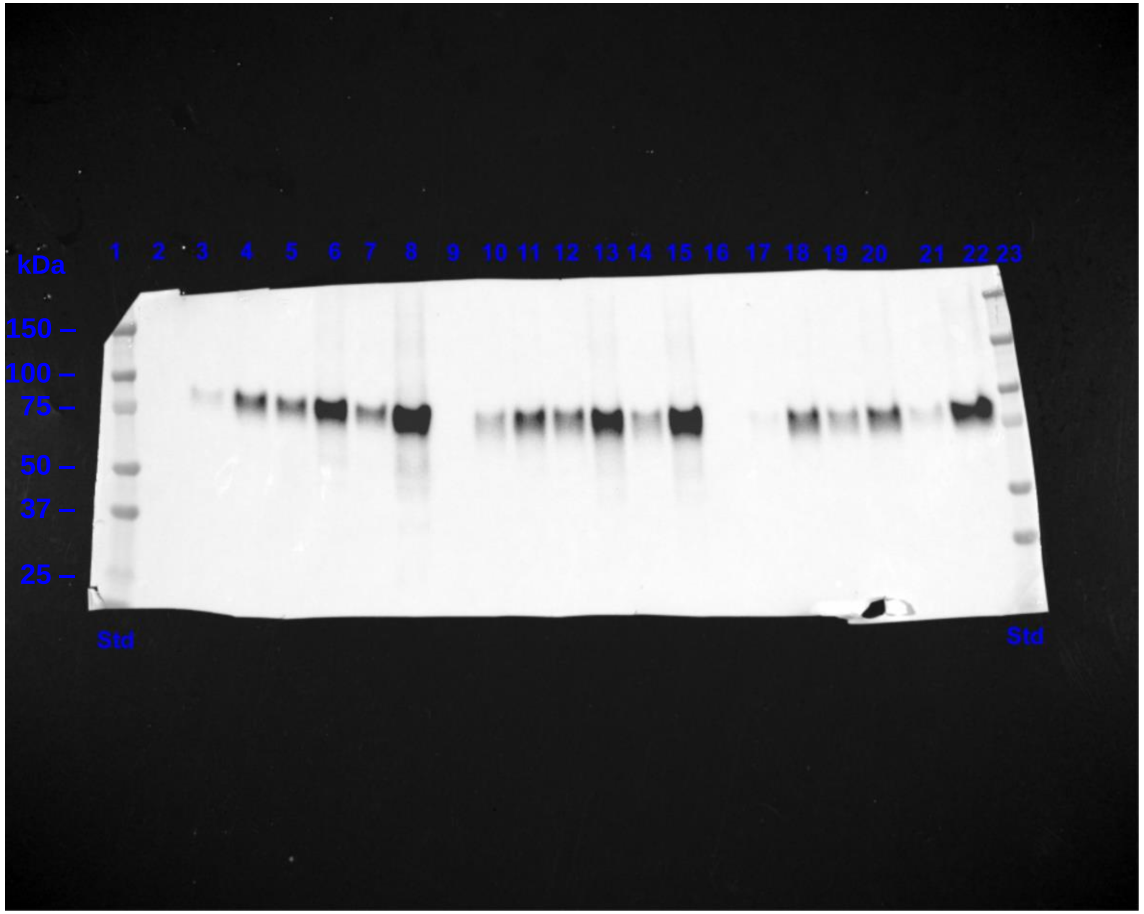

Fig.6C Mdm2 synthesis with WT and ΔSSA1 extracts (trial 2)

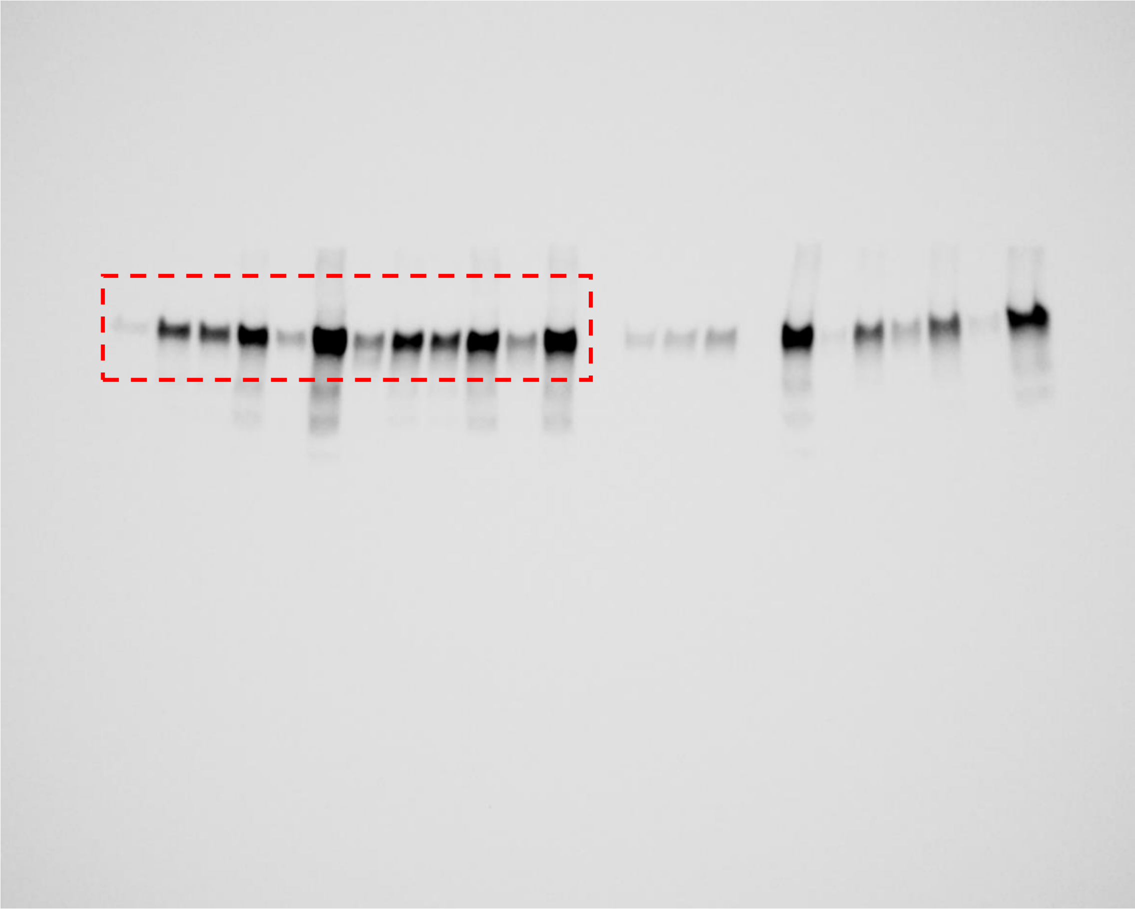

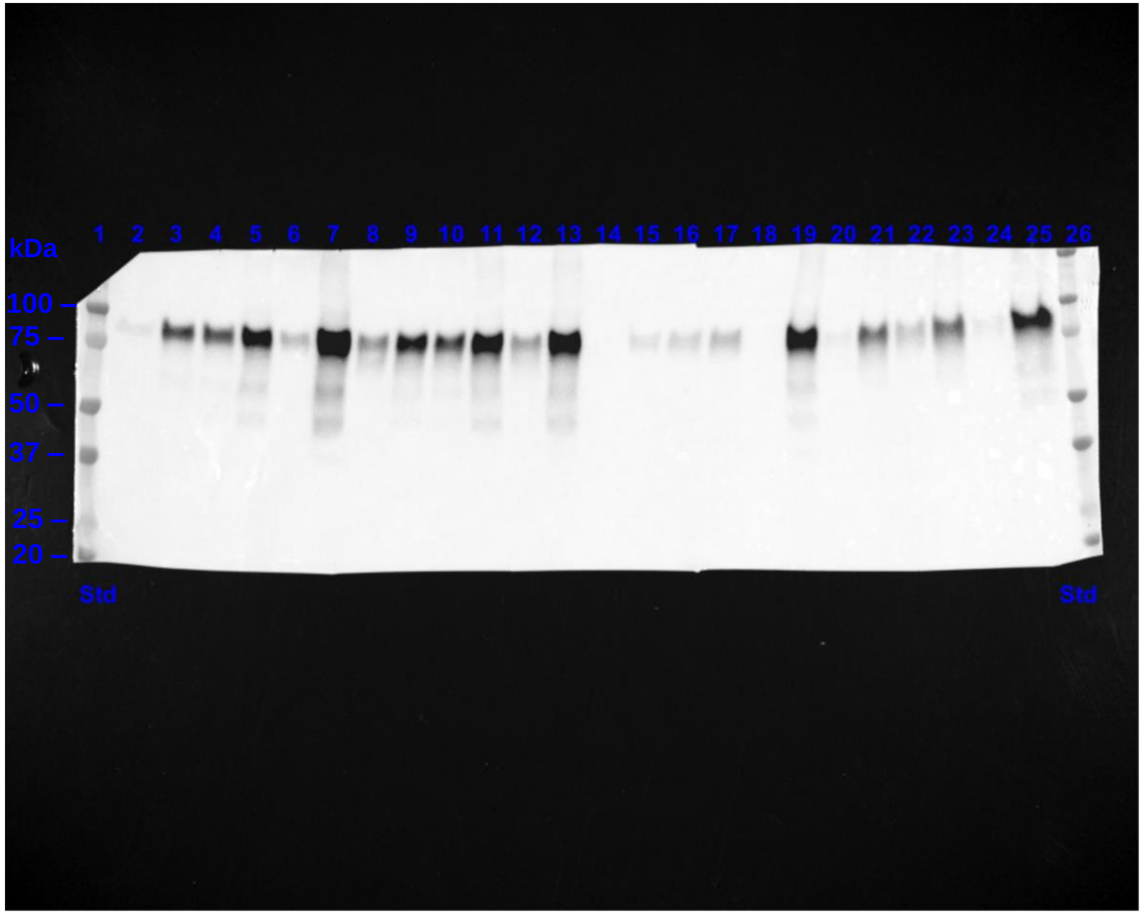

Supplement: CB-005-D4CB00004H-s005 [file CB-005-D4CB00004H-s005.pdf]
